# Cosolvent‐Regulated Weakly Solvating Locally Concentrated Ionic Liquid Electrolyte for Long‐Life Lithium Metal Batteries at Low Temperatures

**DOI:** 10.1002/smsc.202500590

**Published:** 2026-02-27

**Authors:** Lei Xu, Bing Ding, Chong Xu, Miao Xu, Zengjie Fan, Peng Song, Jie Wang, Xiaogang Zhang, Yusuke Yamauchi

**Affiliations:** ^1^ Jiangsu Key Laboratory of Electrochemical Energy‐Storage Technologies College of Materials Science and Technology Nanjing University of Aeronautics and Astronautics Nanjing China; ^2^ Shanghai Institute of Space Power‐Sources/State Key Laboratory of Space Power‐Sources Shanghai China; ^3^ Australian Institute for Bioengineering and Nanotechnology (AIBN) The University of Queensland Brisbane Australia; ^4^ Department of Materials Process Engineering Graduate School of Engineering Nagoya University Nagoya Japan; ^5^ Department of Chemical and Biomolecular Engineering Yonsei University Seoul Republic of Korea

**Keywords:** ionic liquids, lithium metal batteries, locally concentrated electrolytes, low temperature, weak solvation

## Abstract

Lithium metal batteries (LMBs) are attractive next‐generation high‐energy‐density systems, but their operation at low temperatures is hindered by sluggish ion transport and unstable interfaces, which trigger dendrite formation and poor Coulombic efficiency. Herein, we introduce monofluorobenzene (FB) as the weakly solvated cosolvent to modulate the liquidity and microstructure of locally concentrated ionic liquid electrolytes to address these challenges. The weak interaction between FB and Li^+^ as well as organic cations not only weakens the strong coordination of Li^+^‐anion but also loosens the dense ionic aggregation structure, enabling fast Li^+^ transport in both the bulk and interface. Simultaneously, it drives preferential anion decomposition at the interface, yielding a robust inorganic‐rich solid electrolyte interphase. The optimized electrolyte enables stable lithium plating/stripping for 4000 h with low overpotential and achieves outstanding cycling performance in Li||NCM93 cells, maintaining stable cycling for over 500 cycles at −20°C. This work establishes a practical electrolyte design strategy for enabling long‐cycling, low‐temperature LMBs.

## Graphical abstract

Monofluorobenzene‐regulated weakly solvating ionic liquid electrolyte enhances Li^+^ transport and drives inorganic‐rich solid electrolyte interphase formation, enabling stable long‐cycle operation of lithium metal batteries at low temperatures. The optimized electrolyte achieves outstanding cycling performance in Li||NCM93 cells, maintaining stable cycling for over 500 cycles at −20°C.

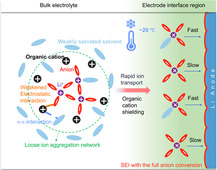

## 1 Introduction

Lithium metal anodes (LMAs) are regarded as the most promising candidates for next‐generation high‐energy‐density batteries because of their ultrahigh theoretical capacity (3860 mAh g^−1^) and low redox potential (−3.04 V vs. SHE) [1, 2]. Meanwhile, nickel‐rich layered oxide cathodes (LiNi_*x*_Co_*y*_Mn*_z_* O_2_, NCM, *x* + *y* + *z* = 1, *x* ≥ 90%) deliver high practical specific capacities (≈200 mAh g^−1^) together with elevated operating voltages (≈3.8 V vs*.* Li^+^/Li) [3]. Pairing LMAs with Ni‐rich NCM cathodes therefore offers the potential to maximize the energy density of lithium metal batteries (LMBs) [4, 5]. However, such configurations impose stringent demands on electrolyte stability. Without robust interfacial protection, both the lithium anode and Ni‐rich NCM cathode exhibit poor compatibility with conventional electrolytes, leading to severe side reactions and rapid performance degradation [6, 7]. At low temperatures (<−10°C), decreased ionic conductivity and increased Li^+^ desolvation barriers aggravate uneven lithium deposition and dendrite growth, further accelerating interfacial degradation and raising safety risks [8, 9]. Therefore, the development of electrolytes capable of maintaining interfacial stability under low‐temperature conditions is critical for the practical deployment of high‐energy LMBs.

Electrolyte engineering has emerged as an effective strategy for enabling low‐temperature operation, as electrolytes not only mediate Li^+^ transport but also regulate the formation of the solid electrolyte interphase (SEI) layer [10, 11]. Room‐temperature ionic liquid electrolytes (ILEs) are particularly attractive because of their negligible volatility, excellent thermal stability, and wide electrochemical window [12, 13]. Nevertheless, their intrinsically high viscosity and sluggish Li^+^ transport become even more problematic at subambient temperatures [14, 15, 16]. To overcome these challenges, locally concentrated ionic liquid electrolytes (LCILEs), formed by introducing inert nonsolvating diluents into ILEs, have been developed [17, 18, 19, 20, 21, 22, 23]. Although LCILEs extend the operating temperature to as low as −60°C, their performance remains inadequate. For example, Li||LiNi_0.6_Co_0.2_Mn_0.2_O_2_ cells cycled at −30°C deliver only ≈0.64 mAh cm^−2^ at 0.085 mA cm^−2^, far below the requirements for high‐capacity LMBs [20].

The solvation structure of LCILEs plays a pivotal role in determining Li^+^ desolvation kinetics at electrode interfaces and the corresponding interfacial chemistry, directly impacting dendrite suppression under low‐temperature operation [24, 25, 26]. Despite substantial progress, the solvation structure of LCILE under extreme conditions remains poorly understood [14, 25]. In particular, the interactions between organic cations and diluents, as well as between anions and diluents, have not yet been clearly resolved [27]. Gaining molecular‐level insight into these interactions is essential for guiding the rational design of low‐temperature electrolytes. Furthermore, the high cost of commonly used ionic liquids (e.g., 1‐methyl‐1‐propyl pyrrolidinium bis(fluorosulfonyl)imide, Pyr_13_FSI) and fluoroether diluents (e.g., 1,1,2,2‐tetrafluoroethyl‐2,2,3,3‐tetrafluoropropyl ether, TTE) continues to hinder the scalability and commercial viability of LCILE [28, 29, 30].

Herein, monofluorobenzene (FB) is introduced as a cosolvent in a LiFSI‐(1‐ethyl‐3‐methylimidazolium bis(fluorosulfonyl)imide) (EmimFSI) electrolyte for low‐temperature LMBs. FB, as a weakly solvating cosolvent, is utilized to enhance the liquidity and microstructure of LCILEs. The weak interaction of FB with Li^+^ and Emim^+^ reduces the strong coordination between Li^+^–FSI^−^, loosening the dense ionic aggregation structure and enabling rapid Li^+^ transport in both the bulk phase and at interfaces. At the lithium anode/electrolyte interface, preferential anion decomposition generates a robust, inorganic‐rich SEI. As a result, the optimized LCILE enables highly stable lithium plating/stripping with low overpotentials and delivers exceptional performance in Li||LiNi_0.93_Co_0.035_Mn_0.035_O_2_ (NCM93) full cells.

## 2 Results and Discussion

**FIGURE 1:**
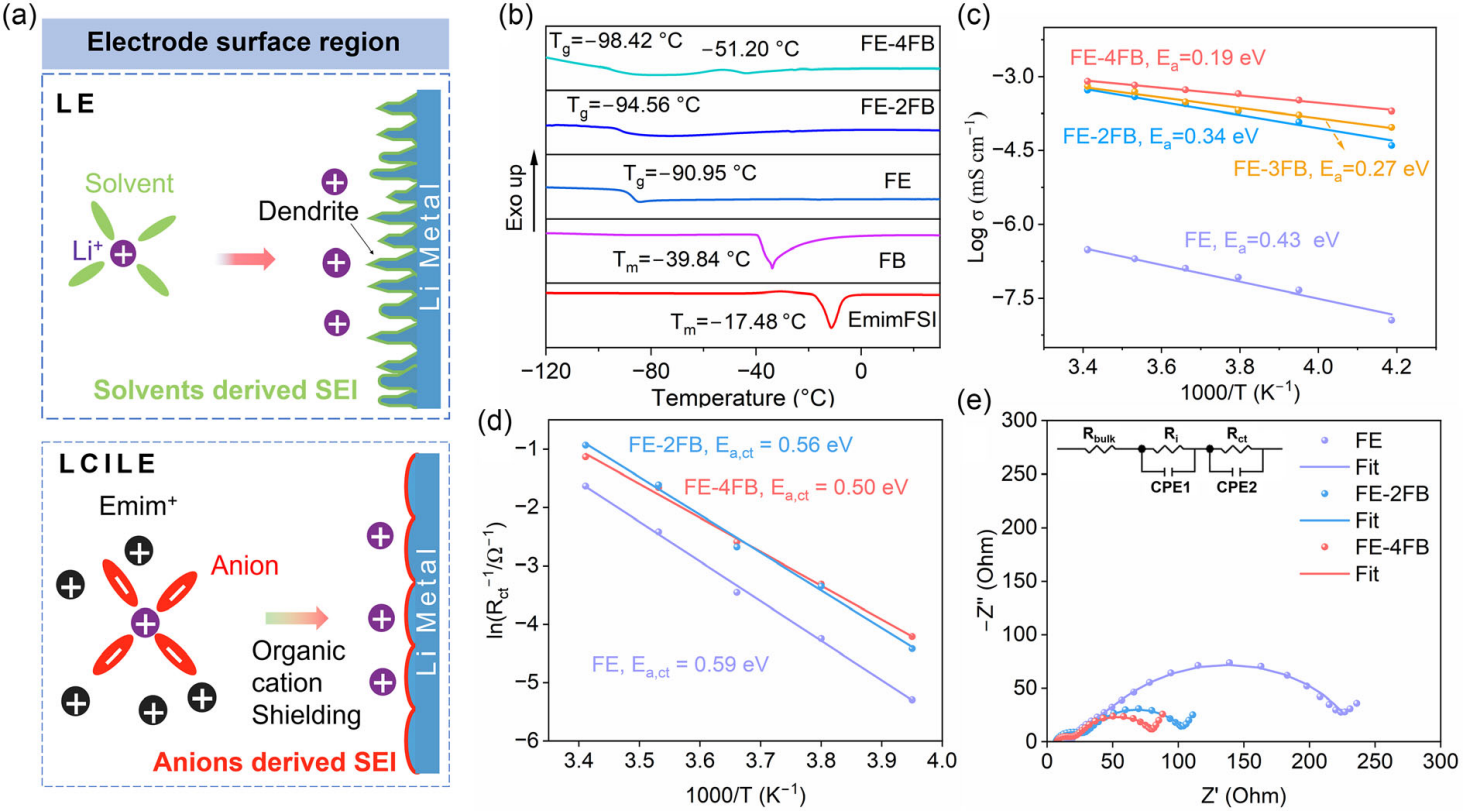
(a) Schematic illustration of the Li^+^ desolvation process at the electrolyte/lithium anode interface in LE (top) and LCILE (bottom). (b) DSC curves of EmimFSI, FB, FE, FE‐2FB, and FE‐4FB. (c) Arrhenius plots of ionic conductivity for the different electrolytes. (d) Arrhenius plot for determining the activation energy of desolvation (*E*_a,ct_). (e) Nyquist plots of Li||Li symmetric cells with electrolytes at −20°C. LCILE = Locally concentrated ionic liquid electrolyte; DSC = differential scanning calorimetry.

Owning its low viscosity and weak dissociation with LiFSI at subzero temperatures, FB is introduced as a cosolvent in LCILEs to reduce viscosity and enhance Li^+^ conductivity at low temperatures. As shown in Figure 1a, the anion‐dominated solvation sheath not only lowers Li^+^ desolvation energy but also promotes the formation of a robust, inorganic‐rich SEI, thereby enabling uniform lithium stripping/plating. To systematically probe the role of FB, LCILEs with LiFSI/EmimFSI/FB molar ratios of 1:2:0, 1:2:2, 1:2:3, and 1:2:4 were prepared and designated as FE, FE‐2FB, FE‐3FB, and FE‐4FB, respectively (Table S1). Differential scanning calorimetry (DSC) was employed to examine their phase‐transition behavior (Figure 1b). The melting points of EmimFSI and FB were −17.48 and −39.84°C, respectively. Upon dissolving LiFSI in EmimFSI (FE electrolyte), no melting temperature (*T*_*m*_) was observed, but a glass transition temperature (*T*_g_) of −90.95°C was detected, in line with prior reports [14]. With increasing FB content, LCILEs exhibited progressively lower *T*_g_ values (–94.56°C for FE‐2FB and –98.42°C for FE‐4FB). In FE‐4FB, an additional small endothermic peak appears at −51.20°C, which primarily results from the high concentration of FB present in FE‐4FB. These results show that FE and FB‐containing LCILEs remain liquid at −50°C.

Because electrolyte viscosity increases significantly at low temperatures, ionic conductivity is usually compromised [31, 32]. The practical fluidity of the electrolyte at −50°C was also visually confirmed (Figure S1). Unlike FE, which showed poor flowability, FB‐containing LCILEs (FE‐2FB to FE‐4FB) maintained higher fluidity without phase separation. However, increasing FB content markedly reduced the viscosity of the LCILEs, owing to its intrinsic low viscosity [33]. When the molar ratio of LiFSI/EmimFSI/FB is 1:2:4, FB content in the electrolyte approaches its maximum solubility (Figure S1a,b). Further increasing the FB ratio to 1:2:5 and 1:2:6 results in noticeable phase separation in the LCILEs (Figure S1c). The viscosity decreased from 507.1 mPa s (FE) to 107.9 mPa s (FE‐2FB) and 12.9 mPa s (FE‐4FB) at −20°C (Figure S2). Consequently, ionic conductivity at −20°C increased from 1.26 × 10^−4^ (FE) to 0.331 mS cm^−1^ (FE‐4FB) (Figure 1c), surpassing that of a conventional liquid electrolyte (LE, 1.0 M LiPF_6_ in DMC/EMC/EC, 0.273 mS cm^−1^, Figure S3). In addition, as revealed from the pulsed‐field gradient nuclear magnetic resonance spectra (PFG‐NMR, Figure S4) [7, 16], the self‐diffusion coefficients of Li^+^, FSI^‐^, and Emim^+^ in FE‐4FB are 1.63, 1.61, and 1.53 times greater, respectively, than those in FE‐2FB. The notably higher Li^+^ self‐diffusion coefficient and higher ionic conductivity suggest enhanced Li^+^ migration efficiency in the FE‐4FB electrolyte. The desolvation energy barrier of lithium ions in FE‐4FB is 0.50 eV, which is significantly lower than that in FE (0.59 eV) (Figure 1d and Figure S5). At −20°C, the charge‐transfer resistance (*R*_ct_) decreases from 199.4 Ω for FE to 67.5 Ω for FE‐4FB, indicating that FB effectively enhances the interfacial Li^+^ desolvation kinetics (Figure 1e and Table S2). Linear sweep voltammetry (LSV) results (Figure S6) revealed wide electrochemical stability windows for all LCILEs, with oxidation onset potentials around ≈4.60 V (vs. Li^+^/Li), indicating strong resistance to high‐voltage decomposition. Contact angle tests (Figure S7) demonstrated improved wettability of LCILEs on polypropylene (PP) separators, suggesting superior interfacial compatibility and faster electrolyte infiltration. Furthermore, substituting high‐cost LiFSI and EmimFSI with the inexpensive FB cosolvent substantially reduced overall electrolyte cost (Figure S8) [28].

**FIGURE 2:**
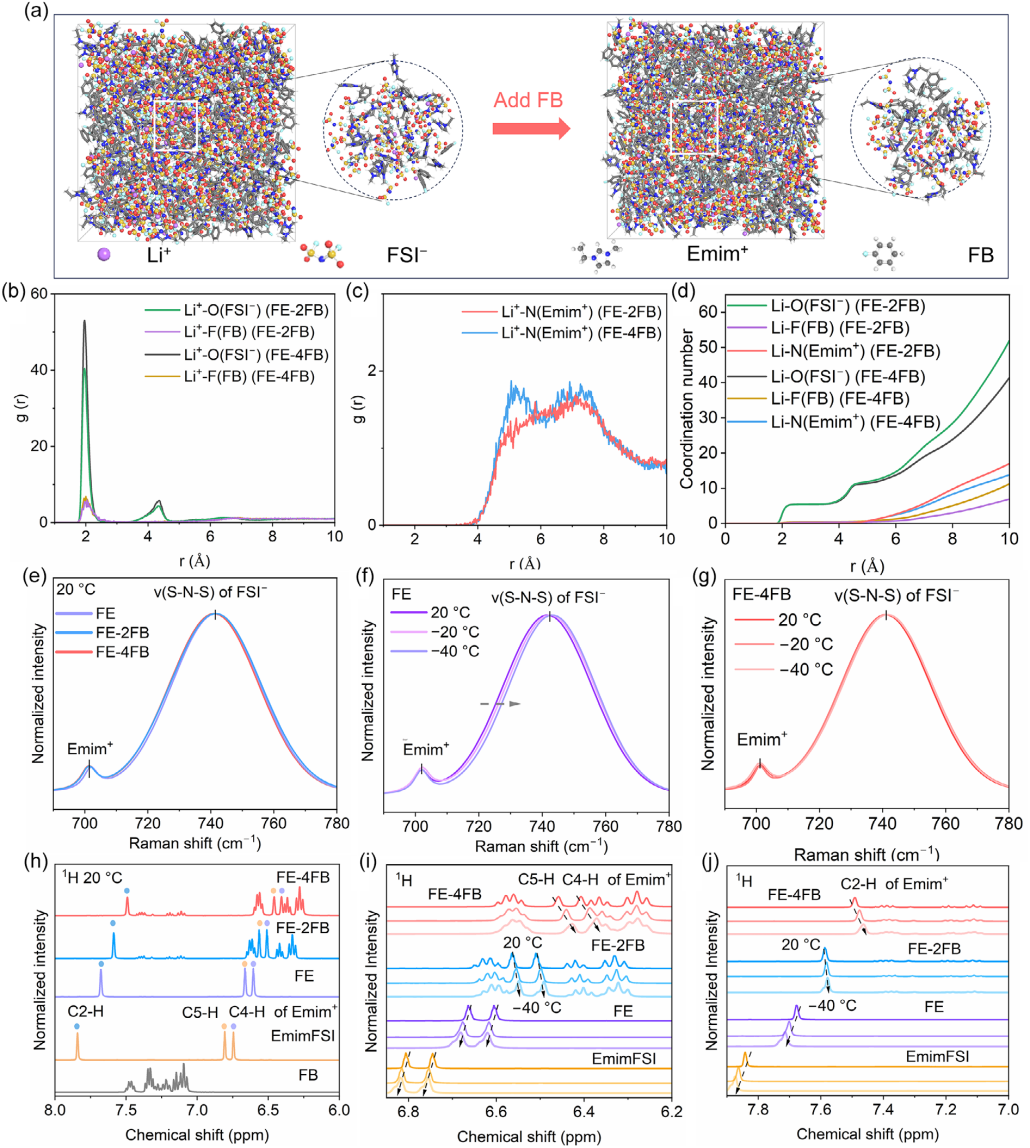
Solvation structure of the electrolytes. (a) Snapshots of the MD simulated boxes of FE‐2FB (left) and FE‐4FB (right) at −20°C. (b, c) RDFs and (d) CNs of Li^+^. (e) Raman spectra of the electrolytes. Temperature‐dependent Raman spectra of (f) FE and (g) FE‐4FB. ^1^H NMR spectra of the electrolytes and their components in the regions of (h) 6.00–8.00 ppm at 20°C, (i) 6.20–6.85 ppm at 20, −20 and −40°C, and (j) 7.00–7.90 ppm at 20, −20 and −40°C. MD = Molecular dynamics; RDFs = radial distribution functions; CNs = coordination numbers.

To analyze the microstructure of LCILEs at −20°C, molecular dynamics (MD) simulations were conducted. Both FE‐2FB and FE‐4FB (Figure 2a) exhibit heterogeneity in their microstructures, characterized by ion aggregation and separate regions formed by FB cosolvent. The radial distribution functions (RDFs) of Li^+^ were employed to elucidate the solvation structure in more detail (Figure 2b,c). Due to the lower volume fraction of ion occupancy in FE‐4FB compared to FE‐2FB, the intensity of the curve corresponding to Li^+^ coordination with ions is enhanced in FE‐4FB. Distinct peaks at 1.96 Å corresponding to Li–O(FSI^−^) and smaller peaks at 2 Å associated with Li–F(FB) were observed, while signals related to Li^+^–N(Emim^+^) only appeared beyond 4.08 Å. This result confirms that Li^+^ coordinates predominantly with FSI^‐^, with minimal interaction with FB, while Emim^+^ resides outside the Li^+^ solvation shell, forming a Li^+^–FSI^‐^–Emim^+^ network structure. Furthermore, the statistical results of coordination number (CN) distributions show that beyond 4.55 Å, FE‐4FB exhibits fewer Emim^+^ and more FB compared to FE‐2FB (Figure 2d). This suggests an increased presence of FB within the Li^+^–FSI^−^–Emim^+^ network. As quantified analysis of the coordination populations (Table S3), the average CN of Li^+^–FSI^−^ in the FE‐2FB (3.523) is higher than that in the FE‐4FB (3.189), while the average CN of Li^+^‐FB in FE‐2FB (0.218) is lower than that in FE‐4FB (0.351). The increase in weakly coordinated Li^+^–FB interactions contributes to a reduction in the strong coordination between Li^+^ and FSI^−^, thereby lowering the desolvation energy barrier of Li^+^ and improving the kinetics of Li^+^ transport at low temperatures.

The temperature‐variable Raman spectroscopy was conducted to demonstrate the evolution of Li^+^ solvation structures in LCILEs at low temperatures. Raman spectra were collected in the 690–780 cm^−1^ region, corresponding to the symmetric stretching vibration (*v*(S–N–S)) of the FSI^‐^ anion (Figure 2e–g and Figure S9) [7]. For neat EmimFSI, the dominant peak at 725 cm^−1^ arises from “free” FSI^−^ weakly coordinated with the bulky, charge‐delocalized Emim^+^ cation, accompanied by a minor peak near 702 cm^−1^ attributable to Emim^+^ [34]. In contrast, upon the introduction of LiFSI in FE and LCILEs, the *v*(S–N–S) peak shifts to 741 cm^−1^, indicative of Li^+^–FSI^−^ coordination. At 20°C, varying the FB content does not induce additional peak shifts. Upon cooling, a slight rightward shift in the peaks of FE and FE‐2FB is observed, while no peak displacement occurs for FE‐4FB. This indicates a weaker coordination of Li^+^–FSI^−^ in FE‐4FB compared to FE and FE‐2FB at low temperatures, which is consistent with the MD simulation results.

Figure 2h‐j and Figure S10 show the 1H NMR spectra of FE, LCILEs, EmimFSI, and FB to investigate the interaction between Emim^+^ and FB. At 20°C, in FE, the Emim^+^ signals shift upfield, indicating weaker Emim^+^–FSI^‐^ coordination relative to neat EmimFSI. Upon FB addition, both Emim^+^ and FB proton peaks exhibit pronounced upfield shifts. Specifically, the aromatic protons of FB shift from 7.05–7.51 ppm to 6.28–6.66 ppm (FE‐2FB) and 6.24–6.60 ppm (FE‐4FB). These shifts originate from *π*–*π* stacking between Emim^+^ and FB (Figure S11) [33, 35, 36, 37], which promote charge transfer from Emim^+^ to the FB aromatic ring, enhancing local electron density and proton shielding [36, 37]. For FB, the FB signals remain essentially unchanged upon cooling. In contrast, both EmimFSI and FE shift downfield with decreasing temperature, consistent with solidification of EmimFSI and reduced fluidity of FE. For FE‐2FB and FE‐4FB, both Emim^+^ and FB peaks shift upfield upon cooling, with FE‐4FB showing a greater high‐field shift than FE‐2FB, reflecting strengthened *π*–*π* interactions. The *π* *–π* stacking interactions increase with higher FB content and lower temperature, reducing the interaction between Emim^+^ and Li^+^–FSI^−^ aggregates, which allows more FB to engage in the Li^+^–FSI^−^–Emim^+^ network. Furthermore, the heterogeneity of the microstructure in LCILEs is also reflected in NMR testing. The Li^+^–FSI^−^–Emim^+^ ionic network containing FB is primarily associated with the peaks observed in the ^1^H NMR spectrum at 6.20–6.70 ppm (Figure 2i). In the diluent region, the mutual solubility of EmimFSI and FB results in a small amount of EmimFSI being present, which corresponds to the peaks in the ^1^H NMR spectrum at 7.08–7.42 ppm (Figure S10b). Collectively, at low temperatures, an increase in FB content leads to more FB participating in the initial solvation shell of Li^+^ and joining the Li^+^–FSI^−^–Emim^+^ network. This results in a looser ionic network that reduces the desolvation energy barrier for Li^+^ and increases the rate of Li^+^ transport.

**FIGURE 3:**
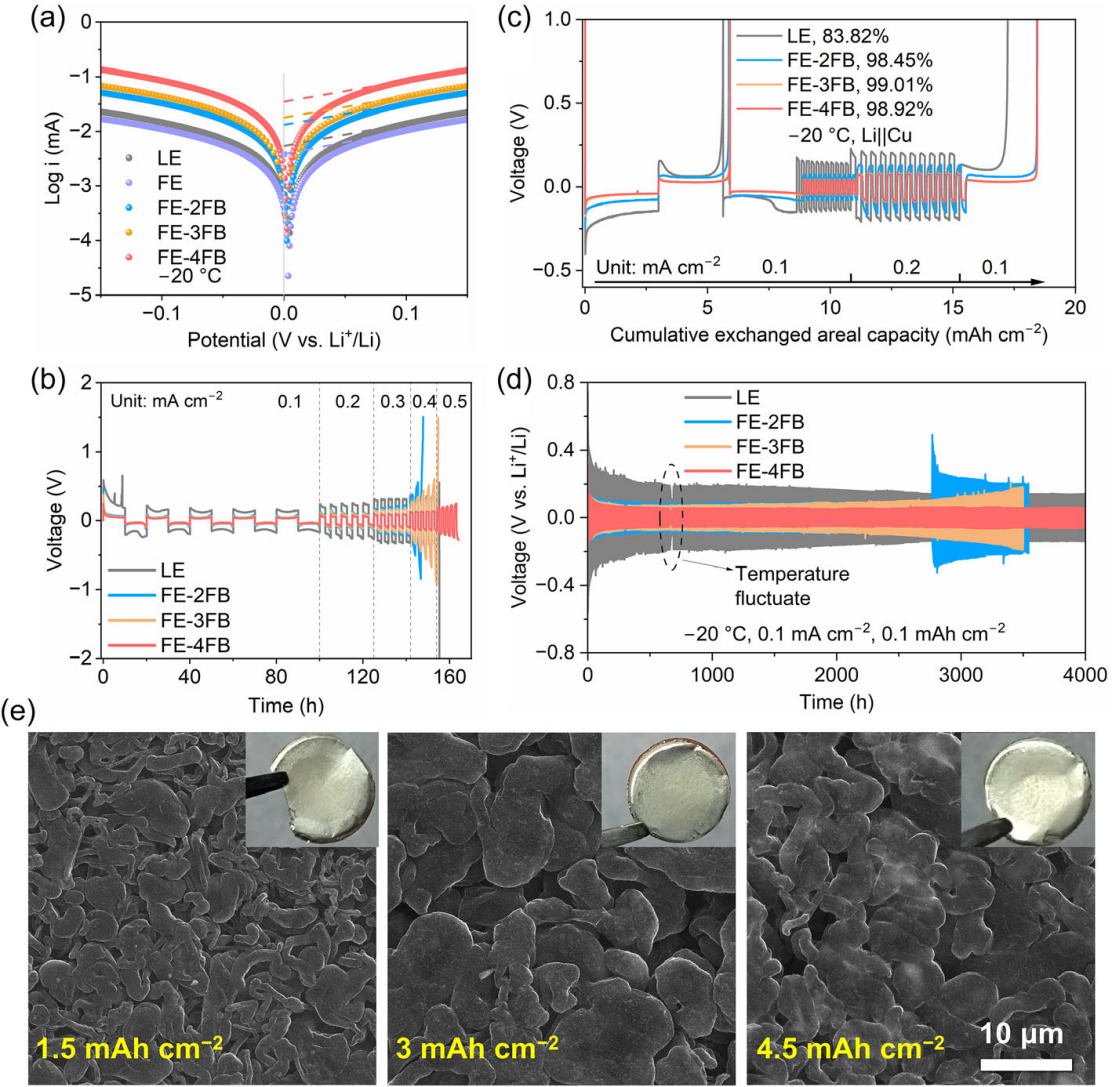
(a) Tafel curves of Li||Li cells. (b) Voltage profiles of Li||Li cells with different electrolytes at −20°C. (c) Voltage profiles of Li||Cu cells at different current densities. (d) Long‐term galvanostatic cycling voltage of Li||Li cells. (e) SEM images of lithium deposited on Cu foil at various deposition capacities for Li||Cu cells with FE‐4FB at 0.1 mA cm^−2^ (inset: corresponding optical photographs of deposited lithium). SEM = Scanning electron microscopy.

To evaluate interfacial kinetics at subzero temperatures, Li||Li symmetric cells and Li||Cu cells were assembled and tested at −20°C. As shown in Figure 3a and Figure S12, increasing FB content in LCILEs significantly enhanced the exchange current density and reduced polarization. In particular, the exchange current density of the cell using FE‐4FB reached 0.0114 mA cm^−2^, nearly seven times higher than that of the LE. Voltage profiles (Figure 3b and Figure S13) further demonstrate the impact of FB addition. After activation for five cycles at 1 mAh cm^−2^ (0.1 mA cm^−2^), cells were cycled at 0.5 mAh cm^−2^ with varied current densities. The Li||FE||Li cell short‐circuited at 0.1 mA cm^−2^ due to poor wettability and sluggish Li^+^ transport. At low current densities (<0.2 mA cm^−2^), all LCILE‐based cells showed comparable polarization, suggesting similar interfacial resistance. At higher current densities, however, FE‐4FB exhibited the lowest overpotential (≈145 mV at 0.4 mA cm^−2^), whereas the LE displayed severe polarization (>400 mV at 0.4 mA cm^−2^) and failed at 0.5 mA cm^−2^. The weak dependence of overpotential on bulk ionic conductivity indicates that concentration polarization is negligible. Instead, polarization primarily originates from the high Li^+^ desolvation barrier at the electrode/electrolyte interface. These results indicate that the desolvation process of Li^+^ at the interface plays a critical role in determining the polarization behavior during lithium stripping and plating [38, 39]. Coulombic efficiency (CE) tests in Li||Cu cell (Figure 3c) corroborated these findings. LCILEs‐based cells delivered CE values exceeding 98%, with FE‐4FB achieving 98.92%, while LE showed only 83.82%. Voltage profiles at 0.2 and 0.2 mAh cm^−2^ further confirmed reduced polarization with increasing FB content (Figure S14), evidencing more reversible lithium deposition.

Long‐term cycling tests of Li||Li cells highlight the durability benefits of FB addition (Figure 3d). The average voltages over 1000–2000 cycles were 175.8, 56.8, 54.1, and 42.0 mV for LE, FE‐2FB, FE‐3FB, and FE‐4FB, respectively (Figure S15). Electrochemical impedance spectroscopy (EIS) after 50 cycles (Figure S16) revealed that the interfacial resistance of LE reached ≈2000.0 Ω for LE, whereas FE‐4FB remained as low as 178.5 Ω, underscoring the role of reduced desolvation energy in facilitating ion transport. In the later cycling period, Li||Li symmetric cells assembled with different electrolytes did not exhibit any short‐circuit phenomena (Figure S17). FE‐2FB showed a sharp voltage rise at ≈2800 h, likely due to electrolyte depletion from lower CE and ionic conductivity. FE‐3FB exhibited a gradual polarization increase, while FE‐4FB maintained stable cycling for over 4000 h, reflecting uniform lithium plating/stripping and interfacial robustness. While LE can achieve stable cycling at low current densities due to the high Li^+^ conductivity, LE exhibits a much higher polarization overpotential, which is not conducive to the energy utilization of LMBs. Overall, these results demonstrate that the addition of FB not only promotes reversible Li deposition but also minimizes interfacial polarization and enhances long‐term cycling stability. Importantly, the interplay among CE, ionic conductivity, and desolvation energy emerges as the key determinant of reliable low‐temperature LMB performance.

To further elucidate lithium deposition/stripping behavior in different electrolytes, the morphologies of lithium deposited on Cu foil at various capacities were examined. In the case of LE, deposition produced uneven, needle‐like dendrites and mossy lithium with a discontinuous porous structure (Figure S18a), consistent with previous studies [40]. By contrast, LCILE‐based electrolytes yielded markedly improved morphologies. At 1.5 mAh cm^−2^, lithium plated from LCILEs consisted of mixed dendritic and bulk‐like domains (Figure S18b,c). With increasing FB content, interfacial kinetics were enhanced, and FE‐4FB produced a dense, compact lithium layer composed of block‐like particles without observable dendrites (Figure 3e). At higher deposition capacities, larger bulk domains and occasional dendrites were observed, yet the overall morphology remained relatively uniform. Electrolytes with lower FB content, such as FE‐2FB, displayed dendrite‐dominated porous morphologies, whereas FE‐4FB consistently maintained compact, dendrite‐free deposits, correlating with its higher CE and superior cycling stability. Surface analysis of Cu substrates further emphasized these differences. FE‐2FB showed incomplete coverage at low deposition capacities, while all LCILE samples avoided the pronounced protrusions characteristic of LE. Therefore, the anion‐rich solvation structure and favorable interfacial chemistry of LCILEs effectively suppress dendrite formation and penetration through the separator under low‐temperature conditions.

**FIGURE 4:**
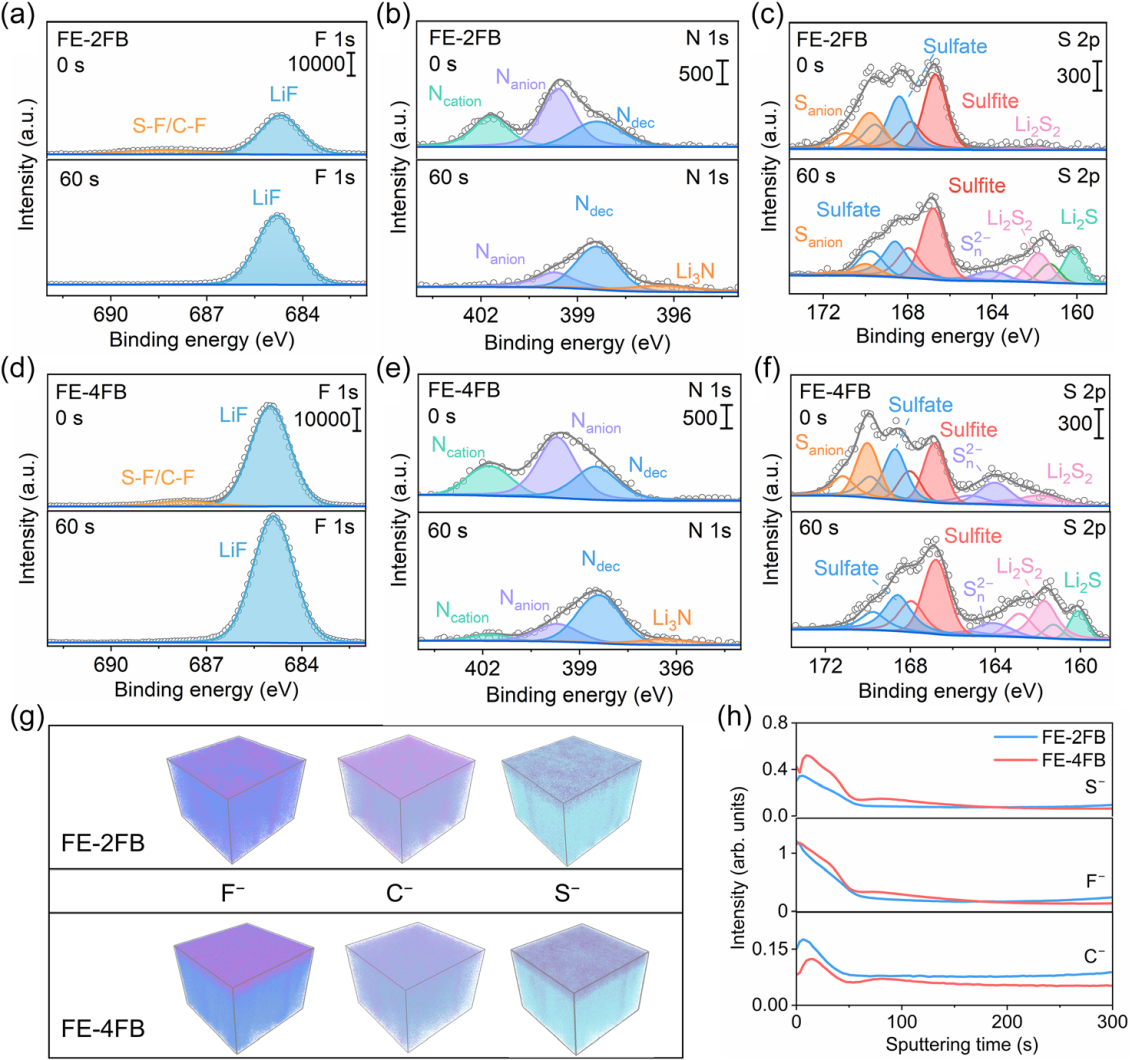
(a–c) F 1s, N 1s, and S 2p XPS spectra of cycled Li metal anodes with FE‐2FB, and (d–f) corresponding spectra with FE‐4FB. (g) 3D elemental distribution maps and (h) corresponding TOF‐SIMS depth profiles of selected fragments for cycled lithium metal surfaces with FE‐2FB and FE‐4FB. TOF‐SIMS = Time‐of‐flight secondary ion mass spectrometry.

To elucidate the composition of the SEI formed in LCILEs at different temperatures, X‐ray photoelectron spectroscopy (XPS) coupled with Ar^+^ sputtering was performed on lithium metal electrodes after cycling at 20 and −20°C (Figures 4a‐f and Figure S19–S20). The N 1s spectrum exhibited four distinct peaks at 401.8, 399.7, 398.4, and 396.4 eV, corresponding to positively charged nitrogen in Emim^+^ (N_cation_), negatively charged nitrogen in FSI^−^ (N_anion_), partially decomposed nitrogen species from Emim^+^/FSI^−^ (N_dec_), and fully decomposed nitrogen forming Li_3_N, respectively [30, 37]. The S 2p spectrum revealed multiple sulfur‐containing products, including intact FSI^−^ (170.0 eV), sulfates (168.6 eV), sulfites (166.6 eV), S—S bonds (S_*n*_^2−^, 164.1 eV), Li_2_S_2_ (161.9 eV), and Li_2_S (160.0 eV), all originating from FSI^−^ decomposition [14, 16, 33]. In the F 1s spectra, peaks at 688.4 and 685.4 eV were assigned to S–F/C–F moieties and LiF, respectively [41, 42]. The C 1s spectra showed contributions from C—C/C=C (284.8 eV), overlapping C=N and C—O bonds (286.4 eV), and C=O (288.4 eV) [14, 41]. At 20°C, compared to FE‐2FB, the cycled lithium metal surface coupled with FE‐4FB exhibits a higher ratio of C—C/C=C, LiF, N_dec_, and Li_3_N. This enhancement is primarily attributed to the higher content of FB in FE‐4FB and the more complete decomposition of FSI^−^, which contributes to stable cycling of LMBs at 20°C (Figure S19). The surface and depth‐profile XPS results of lithium metal after cycling at −20°C are shown in Figures 4a‐f and Figure S20. In the lithium metal surface, the coexistence of N_cation_ and N_anion_ confirms that both cationic and anionic species contribute to SEI formation. Compared with FE‐2FB, FE‐4FB generated significantly higher amounts of fully decomposed FSI^−^ products (e.g., S_*n*_^2−^ and Li_2_S_2_), indicating deeper anion breakdown. FE‐4FB produced more LiF than FE‐2FB, consistent with the stronger anion decomposition suggested by the higher N_dec_ signal in the N 1s spectrum. Depth profiling revealed clear SEI stratification. After Ar^+^ sputtering, the intensities of organic species (C—C/C=C, C–O/C=N, and N_cation_) decreased, whereas inorganic components such as LiF (F 1s), Li_3_N and N_dec_ (N 1s), and Li_2_S_2_/Li_2_S (S 2p) increased, showing that the inner SEI is enriched in inorganic decomposition products [15, 43]. These inorganic phases, with high mechanical strength and poor electronic conductivity, are beneficial for suppressing parasitic reactions and inhibiting dendrite propagation. Among the electrolytes, FE‐4FB yielded the highest LiF content in the inner SEI, consistent with its enhanced interfacial stability and reversible cycling performance. Complementary time‐of‐flight secondary ion mass spectrometry (TOF‐SIMS) analysis confirmed these findings (Figure 4g,h ). The secondary ion distribution and 3D elemental maps revealed that the SEI formed in FE‐4FB is thicker and contains higher F^−^ and S^−^ concentrations compared with FE‐2FB. With increased etching depth, the contents of F and S decreased while carbon remained relatively low, suggesting that FE‐4FB forms an SEI dominated by LiF and inorganic sulfides at the metal surface. Such an inorganic‐rich SEI effectively protects the lithium anode from dendrite growth and electrolyte degradation, fully consistent with XPS results.

**FIGURE 5:**
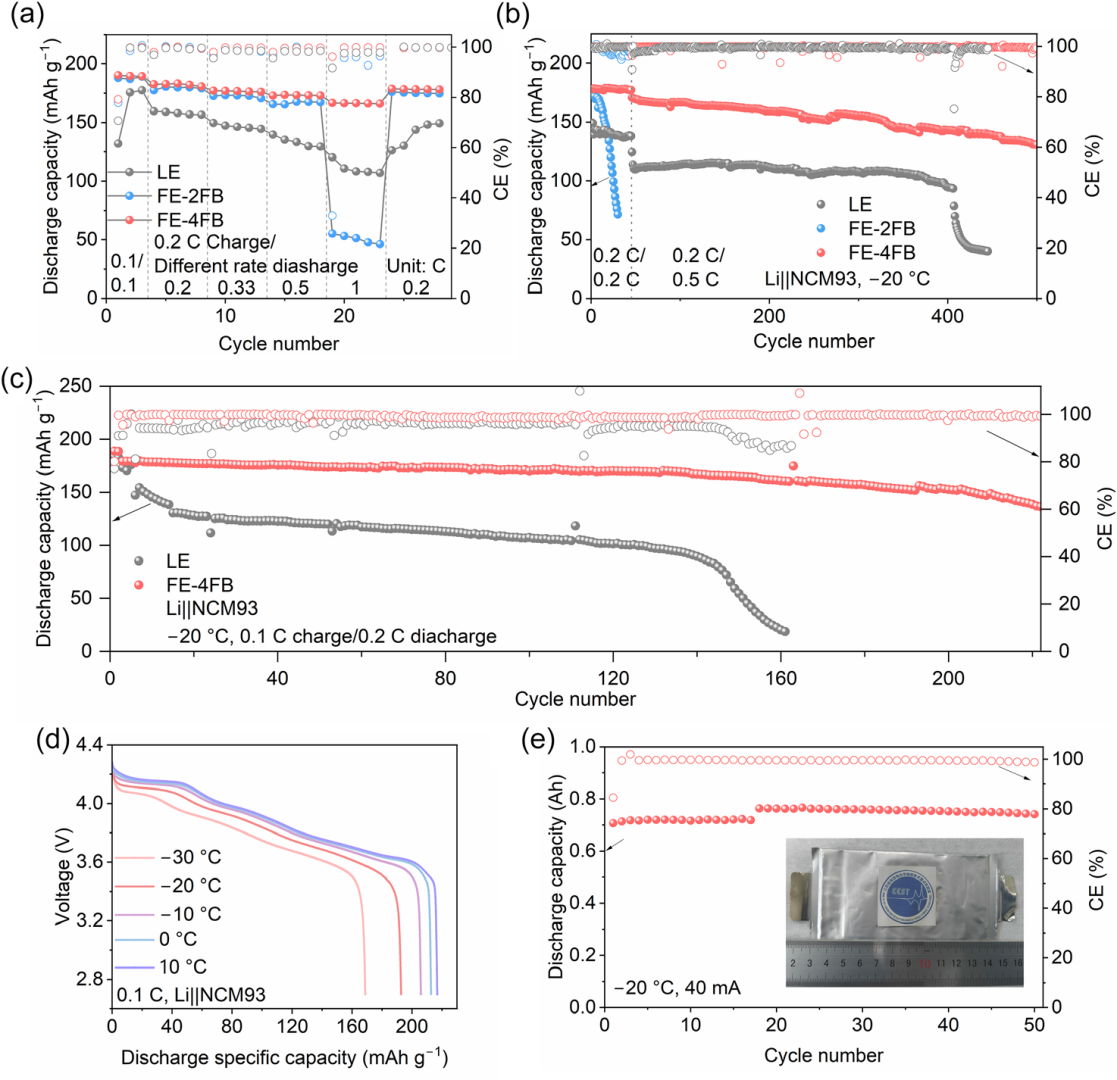
(a) Rate performance of Li||NCM93 cells with LE, FE‐2FB, and FE‐4FB electrolytes at −20°C. (b) Corresponding cycling performance after the rate tests. (c) Cycling stability of Li||NCM93 cells with LE and FE‐4FB electrolytes at −20°C. (d) Temperature‐dependent capacity–voltage profiles of Li||NCM93 cells with FE‐4FB. (e) Cycling performance of a pouch cell with FE‐4FB at −20°C (inset: digital photographs of the pouch cell).

The electrochemical performances of LCILEs were first evaluated in Li||NCM93 cells at 20°C. As shown in Figure S21, LCILE‐based cells exhibited excellent rate capability, maintaining discharge capacities over 189.8 mAh g^−1^ at 2 C, whereas the LE‐based cell delivered only 179.8 mAh g^−1^. After 180 cycles, the capacity retentions of cells with LE, FE‐2FB, FE‐3FB, and FE‐4FB electrolytes were 48.91%, 63.51%, 80.63%, and 82.61%, respectively. Notably, the CE of FE‐2FB dropped below 98% by the 113rd cycle, likely due to elevated FSI^−^ concentration and reduced FB content accelerating anodic corrosion of the Al current collector. In contrast, FE‐4FB maintained a CE of 99.8% throughout 180 cycles, confirming that FB addition effectively suppresses current collector corrosion [44]. The low‐temperature performance of LCILEs was further examined in Li||NCM93 cells (cathode loading: 5 mg cm^−2^) at −20°C. The LE cells delivered an initial discharge capacity of 132.0 mAh g^−1^ and a CE of 70.7% (Figure 5a). In comparison, LCILE‐based cells achieved ≈190.0 mAh g^−1^ with CEs exceeding 77.5%. Under rate testing with a constant charge rate and varying discharge rates, LCILEs cells retained higher capacities and exhibited lower polarization (Figure S22). At 1 C, FE‐2FB suffered sharp polarization due to poor ionic conductivity, while LE showed moderate polarization. FE‐4FB delivered the highest discharge capacity (165.9 mAh g^−1^) under these conditions. Long‐term cycling tests (Figure 5b) confirmed these trends. After 50 cycles at 0.2 C followed by 0.5 C cycling, FE‐2FB showed rapid capacity fading upon returning to 0.2 C, and LE failed around the 400th cycle. By contrast, FE‐4FB retained 130.9 mAh g^−1^ after 500 cycles.

The FE‐4FB electrolyte also enabled excellent performance in high‐loading Li||NCM93 cells (10 mg cm^−2^) to achieve cycling. During two formation cycles, the cell exhibited an initial CE of 80.2%, which increased to 99.6% in the subsequent cycle (Figure 5c). When cycled at 0.1 C/0.2 C(charge/discharge), it delivered a capacity of 179.1 mAh g^−1^ and retained 77.4% after 220 cycles, with lower polarization (Figure S23). In contrast, the LE cell declined from 173.1 mAh g^−1^ in the first cycle to 130.4 mAh g^−1^ by the 15th cycle and collapsed near the 140th cycle. Differential capacity (dQ/dV) plots (Figure S24) further revealed a sharper H2–H3 transition for NCM93 in FE‐4FB, with a reduced voltage gap (117.8 mV vs. 192.7 mV for LE), indicating improved phase reversibility at low temperatures. Mechanistic insights were obtained by distribution of relaxation times (DRT) analysis (Figure S25). After 150 cycles, the interfacial impedance (*R*_*i*_) of LE increased due to interfacial side reactions, while the apparent decrease in *R*_ct_ likely resulted from the uneven deposition increasing the effective surface area. In contrast, FE‐4FB maintained consistently low *R*_*i*_ and *R*_ct_ before and after cycling, confirming superior interfacial kinetics and stability. Variable‐temperature tests (Figure 5d) further verified its robustness. Even at −30°C, the Li||NCM93 cell delivered 168.8 mAh g^−1^ at 0.1 C. Finally, a practical pouch cell was assembled using FE‐4FB electrolyte, NCM93 cathode, and lithium anode (60 μm, Table S4). The pouch cell with a cathode mass loading of 10 (mg cm^−2^) exhibited an initial capacity of 760 mAh at −20°C (Figure 5e). During 50 cycles, the cell maintained excellent cycling stability with high CE. With higher cathode mass loading (25 mg cm^−2^) and lower E/C ratio (3.82), the pouch cell achieves a high initial capacity of 2120 mAh at −20°C (Figure S26), demonstrating the practical feasibility of LCILEs for low‐temperature LMBs.

## 3 Conclusions

In conclusion, we present a rational electrolyte design strategy for low‐temperature LMBs by tuning the fraction of a weakly solvating FB cosolvent in LCILEs. This strategy effectively enhances the fluidity of electrolytes at low temperatures while increasing the coordination of FB in the initial solvation shell of Li^+^ and *π* *–π* interactions between FB and cation. This tailors the solvation environment with moderated Li^+^–anion interaction loosening the dense ionic aggregation structure and enabling rapid Li^+^ transport in both the bulk phase and at interfaces. The resulting interfacial chemistry promotes accelerated anion decomposition and yields an inorganic‐rich SEI that stabilizes lithium deposition and enhances CE. Benefiting from these features, the optimized electrolyte delivers exceptional cycling stability in Li||NCM93 cells, retaining high capacity over 500 cycles at −20°C. Moreover, Li||NCM93 pouch cells exhibited robust cycling performances under the same conditions, highlighting the practical promise of this electrolyte design for next‐generation low‐temperature LMBs.

## Supporting Information

Additional supporting information can be found online in the Supporting Information section. **Supporting Fig. S1:** (a) Digital photographs of different electrolytes after storage at −50°C. (b) Corresponding real‐time photographs taken immediately after tilting the samples to assess fluidity. (c) Digital photographs of different electrolytes after storage at 20°C. **Supporting Fig. S2:** Viscosity of different electrolytes measured at 20°C and −20°C. **Supporting Fig. S3:** (a) Arrhenius plots of LE. (b) Ionic conductivities of different electrolytes at −20°C. **Supporting** **Fig. S4:** Self‐diffusion coefficients of the ions in the electrolytes measured via PFG‐NMR. **Supporting** **Fig. S5:** Nyquist plots of Li||Li symmetric cells with (a) FE, (b) FE‐2FB, and (c) FE‐4FB at different temperatures. **Supporting Fig. S6:** (a) LSV curves of FE‐2FB, FE‐3FB, FE‐4FB, and LE electrolytes at room temperature (RT). (b) LSV curves of LE at low temperature. **Supporting Fig. S7:** Contact angle measurements of electrolytes on PP separator: (a) LE, (b) FE, (c) FE‐2FB, (d) FE‐3FB, and (e) FE‐4FB. (f) Bar chart comparing the contact angles of different electrolytes. **Supporting Fig. S8:** (a) Comparison of market prices of current fluorides. (b) Concentrations of FSI^−^, Emim^+^, and FB in LCILEs with varying FB content. **Supporting Fig. S9:** (a) Raman spectrum of EmimFSI at 20°C. (b) Temperature‐dependent Raman spectra of FE‐2FB at 20°C, −20°C, and −40°C. (c) Temperature‐dependent Raman spectra of FB at 20°C, −20°C, and −40°C. **Supporting Fig. S10:** 1D ^1^H NMR spectra of (a) FB in the 6.70–7.60 ppm at 20°C, −20°C, and −40°C and (b) FB, FE‐2FB, FE‐4FB in the 7.00–7.60 ppm region at 20°C. (c) Chemical structure of Emim^+^ with the C2–H, C4–H, and C5–H positions marked. **Supporting Fig. S11:** The optimized *π*–*π* stacking modes of FB with Emim^+^ and the corresponding binding energy (N, F, C, and H atoms are represented by blue, green, gray, and white spheres, respectively). **Supporting Fig. S12:** Comparison of exchange current densities of lithium anode in different electrolytes. **Supporting Fig. S13:** Voltage profiles of a Li||Li symmetric cell with FE electrolyte. **Supporting Fig. S14:** Voltage–capacity curves of Li||Cu cells with FE‐2FB, FE‐3FB, FE‐4FB, and LE electrolytes. **Supporting Fig. S15:** Average voltages Li||Li cells with different electrolytes between 1000^th^ and 2000^th^ cycles shown in Figure 3d. **Supporting Fig. S16:** Nyquist plots of Li||Li cells with different electrolytes after 50 cycles at 0.1 mA cm^−2^ with a cycling capacity of 0.1 mAh cm^−2^ at −20°C. **Supporting Fig. S17:** The particle enlarged figure of Figure 3d. **Supporting Fig. S18:** SEM images of lithium deposits on Cu foil at various deposition capacities for Li||Cu cells with different electrolytes at 0.1 mA cm^−2^ (inset: corresponding digital photographs of lithium deposits on Cu foil): (a) LE, (b) FE‐2FB, and (c) FE‐3FB. **Supporting Fig. S19:** (a) C 1s, (b) N 1s, (c) F 1s and (d) S 2p XPS spectra of cycled lithium metal surface with FE‐2FB and FE‐4FB electrolytes at 20°C. **Supporting Fig. S20:** C 1s XPS spectra of the cycled lithium metal surface with (a) FE‐2FB and (b) FE‐4FB electrolytes at −20°C. **Supporting Fig. S21:** Electrochemical performance of Li||NCM93 cells (NCM93 loading: 10 mg cm^−2^) with FE‐2FB, FE‐3FB, FE‐4FB, and LE electrolytes at room temperature: (a) rate performances and (b) cycling performance. **Supporting Fig. S22:** Charge/discharge voltage profiles of cells using (a) LE, (b) FE‐2FB, and (c) FE‐4FB electrolytes under the cycling conditions described in Figure 5a. **Supporting Fig. S23:** Charge/discharge voltage profiles of cells using (a) LE and (b) FE‐4FB under the cycling conditions described in Figure 5c. **Supporting Fig. S24:** dQ/dV curves of LE and FE‐4FB in the first cycle after activation under the testing conditions described in Figure 5c. **Supporting Fig. S25:** DRT profiles of cell with (a) LE and (b) FE‐4FB electrolyte after activation and after 150 cycles under the testing conditions described in Figure 5c. **Supporting** **Fig. S26:** Charge/discharge voltage profile of a pouch cell with FE‐4FB at −20°C. **Supporting Table S1:** Compositions of the electrolytes. **Supporting Table S2:** Ohmic impedance (*R*_bulk_), interfacial impedance (*R_i_*), and *R*_ct_ of the Li||Li cells according to EIS in Figure 1e. **Supporting Table S3:** Computed CNs of Li^+^ with FSI^−^ and FB in FE‐2FB and FE‐4FB. **Supporting** **Table S4:** Cell parameters of the Li||NCM93 pouch cell. **:** At 20 and −50°C, FE, FE‐2FB, FE‐3FB, and FE‐4FB electrolytes all maintain a homogeneous phase (Figure S1a,b). In contrast, FE‐5FB and FE‐6FB exhibit pronounced liquid–liquid phase separation at 20°C (Figure S1c). Raman analysis focused on the 690–780 cm^−1^ region, corresponding to the symmetric stretching vibration (*v*(S–N–S)) of the FSI^−^ anion. Raman analysis focused on the 750–770 cm^−1^ region, corresponding to FB (Figure S9a). At 20°C, increasing FB content does not affect the peak displacement of LCILEs (Figure 2e), indicating that FB interference can be ignored. As temperature decreases, low FB content FE (Figure 2f) and FE‐2FB (Figure S9b) primarily show a rightward shift in the 710–740 cm^−1^ region, which does not overlap with the FB peak, suggesting that this shift is unrelated to FB. Additionally, the peak of FB shifts to the right as temperature decreases (Figure S9c). In contrast, no peak displacement is observed for high FB content FE‐4FB (Figure 2g). This further indicates that low‐temperature peak displacements in the electrolyte are unrelated to FB. These features support using this region to analyze the Li^+^ solvation structure. The binding energy results from the two *π*–*π* stacking modes indicate the presence of this weak interaction. Additionally, the binding energy of *π*–*π* stacking mode 1 is similar to that of mode 2, suggesting that both stacking modes coexist in the electrolyte. Compared to FE‐2FB, FE‐4FB exhibits a higher ratio of C‐C/C=C, LiF, Ndec, and Li_3_N on the lithium metal surface. This enhancement is primarily attributed to the higher content of FB in FE‐4FB and the more complete decomposition of FSI^−^, which contributes to stable cycling of LMBs at room temperature, consistent with results observed at low temperatures. Additionally, the N 1s and S 2p spectra indicate that Li_3_N and Li_2_S are present in significantly higher proportions within nitrogenous and sulfurous substances than in low‐temperature SEI layers (Figure 4a–f). This suggests that low temperatures severely hindered the conversion of FSI^−^. Additionally, all signals from materials are markedly lower than those observed under low‐temperature conditions. This indicates that the inorganic‐rich SEI layer formed at room temperature substantially enhances interfacial stability and effectively suppresses continuous decomposition of the electrolyte at the interface.

## Author Contributions

**Yusuke Yamauchi:** supervision (lead), writing – review & editing (supporting). **Lei Xu:** data curation (lead), investigation (lead), writing – original draft (lead). **Bing Ding:** conceptualization (lead), project administration (lead), supervision (equal), writing – review & editing (equal). **Chong Xu:** data curation (supporting), writing – original draft (supporting). **Miao Xu:** conceptualization (supporting), project administration (supporting). **Zengjie Fan:** data curation (supporting), investigation (supporting). **Peng** **Song:** data curation (supporting), investigation (supporting). **Jie Wang:** funding acquisition (supporting), project administration (supporting), writing– review & editing (lead), **Xiaogang Zhang:** funding acquisition (equal), project administration (equal), supervision (equal).

## Funding

This study was supported by National Key Research and Development Program of China (2024YFB2408600, 2022YFE0109400), Research Fund of State Key Laboratory of Mechanics and Control for Aerospace Structures (MCAS‐I‐0425G05), Postgraduate Research & Practice Innovation Program of Jiangsu Province (KYCX24_0573), Australian Research Council (DP240100961), ARC Laureate Fellowship (FL230100095), ARC DECRA Fellowship (DE2020003363), and JST‐ERATO Yamauchi Materials Space‐Tectonics Project (JPMJER2003).

## Conflicts of Interest

The authors declare no conflicts of interest.

## Supporting information

Supplementary Material

## Data Availability

The data that support the findings of this study are available from the corresponding author upon reasonable request.
