# Supplementary material for: Cosolvent‐Regulated Weakly Solvating Locally Concentrated Ionic Liquid Electrolyte for Long‐Life Lithium Metal Batteries at Low Temperatures
Source: Small Sci. 2026 Feb 27;6(3):e202500590. doi: 10.1002/smsc.202500590 (PMC12955906; doi:10.1002/smsc.202500590)
Supplement: Supplementary file 1 — Supplementary Material [file SMSC-6-e202500590-s001.pdf]

Supporting Information

Cosolvent-Regulated Weakly Solvating Locally Concentrated Ionic Liquid Electrolyte for Long-Life Lithium Metal Batteries at Low Temperatures

Lei Xu, Bing Ding*, Chong Xu, Miao Xu, Zengjie Fan, Peng Song, Jie Wang*, Xiaogang Zhang*, Yusuke Yamauchi*

L. Xu, B. Ding, C. Xu, Z. Fan, P. Song, X.G. Zhang
Jiangsu Key Laboratory of Electrochemical Energy-Storage Technologies, College of Materials Science and Technology, Nanjing University of Aeronautics and Astronautics, Nanjing 210016, China
E-mail: bingding@nuaa.edu.cn; azhangxg@nuaa.edu.cn

M. Xu
Shanghai Institute of Space Power-Sources/State Key Laboratory of Space Power-Sources, Shanghai 200233, China

J. Wang, Y. Yamauchi
School of Chemical Engineering, Australian Institute for Bioengineering and Nanotechnology (AIBN), The University of Queensland, Brisbane, Queensland 4072, Australia
E-mail: uqjwan72@uq.edu.au; y.yamauchi@uq.edu.au

Y. Yamauchi
Department of Materials Process Engineering, Graduate School of Engineering, Nagoya University, Nagoya 464-8603, Japan
Department of Chemical and Biomolecular Engineering, Yonsei University, Seodaemun-gu, Seoul 03722, Republic of Korea

Keywords: lithium metal batteries, low temperature, ionic liquids, locally concentrated electrolytes, weak solvation

Experimental Section

Materials preparation: LiFSI (DodoChem Co., Ltd.) and EmimFSI (> 99%, Zhejiang Ldet Energy Technology Development Co., Ltd.) were dried under vacuum (~10^{-7} mbar) at 60 °C for 72 h prior to use. Monofluorobenzene (FB, J&K Scientific, 99%) was dried with molecular sieves. Electrolytes with LiFSI:EmimFSI:FB molar ratios of 1:2:0, 1:2:2, 1:2:3, 1:2:4, 1:2:5 and 1:2:6 were designated as FE, FE-2FB, FE-3FB, FE-4FB, FE-5FB and FE-6FB, respectively. All electrolyte formulations were prepared in an argon-filled glovebox (H_2O and O_2 < 0.01 ppm). A commercial reference electrolyte (LE) consisting of 1.0 M $LiPF_6$ in a 1:1:1 (v/v/v) mixture of dimethyl carbonate (DMC), ethyl methyl carbonate (EMC), and ethylene carbonate (EC) was used for comparison. $LiNi_{0.93}Co_{0.035}Mn_{0.035}O_2$ (NCM93, GEM Co., Ltd.) cathodes with mass loading of 5 and 10 mg cm^{-2} were prepared by slurry casting. The slurry was prepared by mixing NCM93, Super P conductive carbon, and polyvinylidene fluoride (PVDF) binder in a mass ratio of 98:1:1 using N-methyl-2-pyrrolidone (NMP, >99%, Sigma Aldrich) as the solvent. The resulting slurry was cast onto aluminum foils and dried to obtain cathode electrodes. Li discs with a thickness of 500 μm and ultra-thin lithium foil with a thickness of 60 μm were obtained from Tianjin Zhongneng Lithium Industry Co., Ltd.

Materials Characterization: Electrolyte viscosity at −20 and 20 °C was measured using an NDJ-5S viscometer (Shanghai Fangrui Instrument Co., Ltd.). Differential scanning calorimetry (DSC) was carried out on a 204F1 Phoenix calorimeter under nitrogen from −120 to 30 °C at a heating rate of 5 °C min^{-1}. Wettability was evaluated by contact angle measurements on polypropylene separators (Celgard 2500) using a JC2000D7M system (Shanghai Zhongchen Digital Technology Equipment Co., Ltd.). Raman spectra were recorded on a LabRAM HR Evolution spectrometer (France) with a 785 nm excitation laser. In the glovebox, the electrolyte was transferred into a 5 mm thin-walled NMR tube (WG-1000-7, Wilmad). A coaxial insert containing deuterated dimethylsulfoxide (DMSO-d6) and acetonitrile-d3 was then placed inside the tube to serve as a locking field and chemical shift reference. The variable temperature ^1H NMR spectra were acquired using a JEOL JNM-ECZ400S NMR spectrometer (JEOL Japan Electronics Co., Ltd), with acetonitrile-d3 employed as both the locking field and chemical shift reference. Self-diffusion experiments for ^1H, ^{19}F, and ^7Li utilized deuterated dimethylsulfoxide (DMSO-d6) as the locking field and chemical shift reference. NMR measurements were conducted at 298 K on a Bruker NEO 600M spectrometer (14.1 T), equipped with a broadband fluorine iProbe for direct observation and a variable temperature unit, without sample rotation. The bipolar pulse longitudinal eddy current delay (BPP-LED) pulse sequence was applied,

along with sinusoidal magnetic field gradients in the z-direction, achieving a maximum intensity of G = 53.5 G cm^{-1}. Lithium deposition morphologies was examined by focused ion beam scanning electron microscope (FIB-SEM, TESCAN LYRA3 GM). The spatial distribution of interfacial elements was analyzed by time-of-flight secondary ion mass spectrometry (TOF-SIMS) employing a Ga^+ ion beam. The chemical composition of SEI layers on lithium metal was investigated by X-ray photoelectron spectroscopy (XPS, ESCALAB Xi+) using Al Kα radiation. The cycled lithium metal samples for TOF-SIMS and XPS measurements were disassembled from the Li||Li symmetric cell after 50 plating/stripping cycles (0.1 mA cm^{-2}, 0.1 mAh cm^{-2} per cycle) in an argon-filled glovebox. The lithium metal samples were then rinsed with dry DMC, and transferred in sealed containers to avoid exposure to air and moisture before XPS analysis.

Electrochemical Measurements: All electrochemical tests were conducted after placing the cells in a temperature-controlled oven and allowing them to equilibrate for more than 2 h at the set temperature. Temperature-dependent ionic conductivity was measured using asymmetric stainless steel (SS||SS) cells. Electrochemical impedance spectroscopy (EIS) was performed using a multichannel electrochemical workstation (Biologic VSP) over a frequency range of 7 MHz to 50 mHz. The ionic conductivity (σ) of the electrolyte was calculated using the following equation:

$$\boldsymbol{\sigma = \frac{l}{R * S}}$$

where R represents the measured impedance, l is the thickness of the separator, and S is the surface area of the SS electrode.

The activation energy barrier ($E_{a,ct}$) for desolvation can be determined by fitting the charge-transfer resistance (R_{ct}) values of Li||Li symmetric cells according to the Arrhenius equation[S1,S2]:

$$\frac{1}{R_{ct}} = A_0 e^{-\frac{E_{ct}}{RT}}$$

where R, T, A_0, $E_{a,ct}$ and R_{ct} stand for the standard gas constant, the absolute temperature, the pre-exponential constant, the activation energy of desolvation and the charge-transfer resistance, respectively.

Electrochemical performance of Li||Cu, Li||NCM93, and Li||Li coin cells were assembled by using Li disc anode and 75 μL of electrolyte was added to each cell. The electrochemical

performances were evaluated using a CT3001A battery testing system (China). CE was evaluated by cycling lithium metal deposited on copper substrates. Prior to cycling, a formation step was conducted by plating 3 mAh cm^{-2} of Li onto a Cu substrate and stripping it at 0.1 mA cm^{-2} to 1 V, minimizing substrate influence. Subsequently, 3 mAh cm^{-2} of Li was deposited onto the Cu electrode at 0.1 mA cm^{-2}, followed by two cycling stages: 11 cycles of plating/stripping 0.1 mAh cm^{-2} at 0.1 mA cm^{-2}, and 11 cycles of 0.2 mAh cm^{-2} at 0.2 mA cm^{-2}. After 22 cycles, the remaining Li was stripped at 0.1 mA cm^{-2} to 1 V. The average CE was calculated as the total stripped capacity (excluding the formation cycle) divided by the total plated capacity.

Theoretical Simulations: All-atom MD simulations were conducted using the GROMACS software package, version 2021.5.[S3–S5] The OPLS-AA force field[S6,S7] was employed to describe the molecule. Subsequently, we randomly placed the optimized molecules into cubic boxes. Molecular dynamics simulations were then performed for these systems. Energy minimization was initially carried out for systems, employing the steepest descent method to address initial contact issues. Followed by a short simulation of 10 ns using the NPT system to make the box fully compressed. Subsequently, A 50 ns simulation was then performed under the NVT ensemble to relax the structure and achieve system equilibrium. The pressure was maintained at P = 1.0 bar using a Berendsen barostat, and the temperature was controlled at 253 K using a velocity-rescale thermostat with a coupling constant of $\tau = 0.1$ ps. Nonbonded interactions were computed with a cutoff of 1.2 nm, and long-range electrostatic interactions were computed using the particle-mesh Ewald summation method. All hydrogen bonds were constrained using the LINCS algorithm.[S8] Simulations were performed with a time step of 2 fs, and the neighbor list was updated every 10 steps.

The configurations of the complex of $Emim^+$-FB were searched using the Molclus[S9] program. The 100 collected structures were optimized using the gfn2-xtb method[S10] with the xtb version 6.6.1 software package[S11] and the optimized structures and corresponding energy data were collected. Structures with similar energies (energy threshold = 1 kcal/mol) and similar structures (geometry threshold = 1 Angstrom) are identified as the same structure. The binding energy was carried out with the Gaussian 16, C01 software package[S12]. The Becke's three-parameter hybrid exchange functionals and the Lee-Yang-Parr correlation functional (B3LYP) was adopted for all calculations in combination with the D3 version of Grimme’s dispersion with Becke-Johnson damping (DFT-D3BJ)[S13]. For geometry optimization and frequency

calculations, the def2-SVP basis set[S14-16] was used. The single point energy calculations were performed with def2-TZVP 10basis set.

Table S1. Compositions of the electrolytes.

Electrolyte	Composition	Molar ratio	Mass ratio
FE	LiFSI:EmimFSI	1:2:0	0.187:0.582:0
FE-2FB	LiFSI:EmimFSI:FB	1:2:2	0.187:0.582:0.192
FE-3FB	LiFSI:EmimFSI:FB	1:2:3	0.187:0.582:0.288
FE-4FB	LiFSI:EmimFSI:FB	1:2:4	0.187:0.582:0.384
FE-5FB	LiFSI:EmimFSI:FB	1:2:5	0.187:0.582:0.480
FE-6FB	LiFSI:EmimFSI:FB	1:2:6	0.187:0.582:0.576

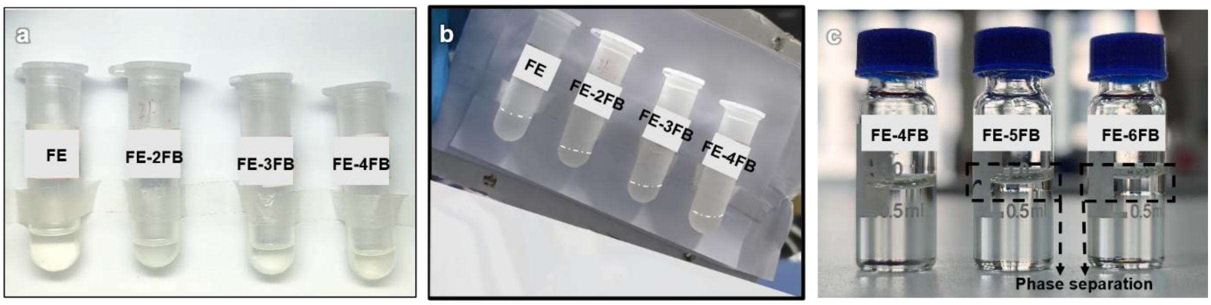

Figure S1. (a) Digital photographs of different electrolytes after storage at −50 °C. (b) Corresponding real-time photographs taken immediately after tilting the samples to assess fluidity. (c) Digital photographs of different electrolytes after storage at 20 °C.

Note 1: At 20 and −50 °C, FE, FE-2FB, FE-3FB, and FE-4FB electrolytes all maintain a homogeneous phase (Figure S1a, S1b). In contrast, FE-5FB and FE-6FB exhibit pronounced liquid–liquid phase separation at 20 °C (Figure S1c).

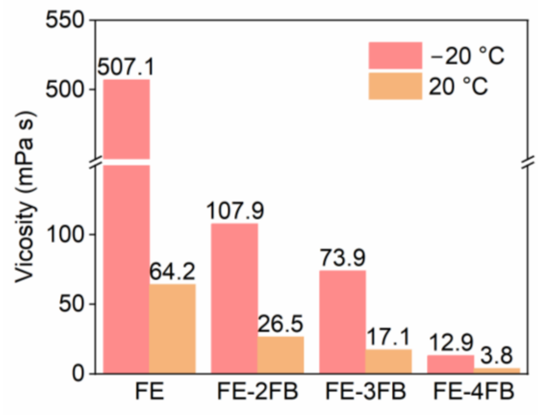

Figure S2. Viscosity of different electrolytes measured at 20 and −20 °C.

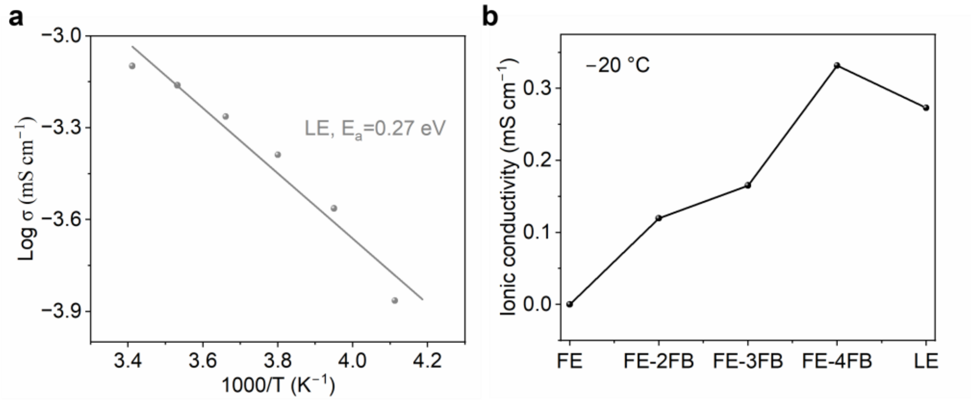

Figure S3. (a) Arrhenius plots of LE. (b) Ionic conductivities of different electrolytes at −20 °C.

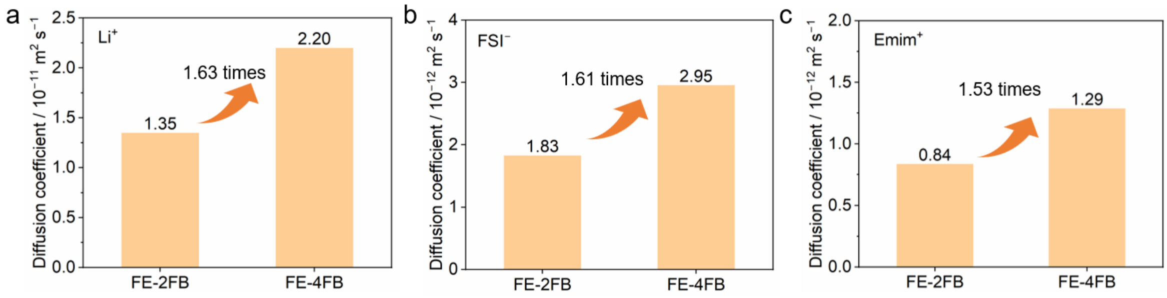

Figure S4. Self-diffusion coefficients of the ions in the electrolytes measured via PFG-NMR.

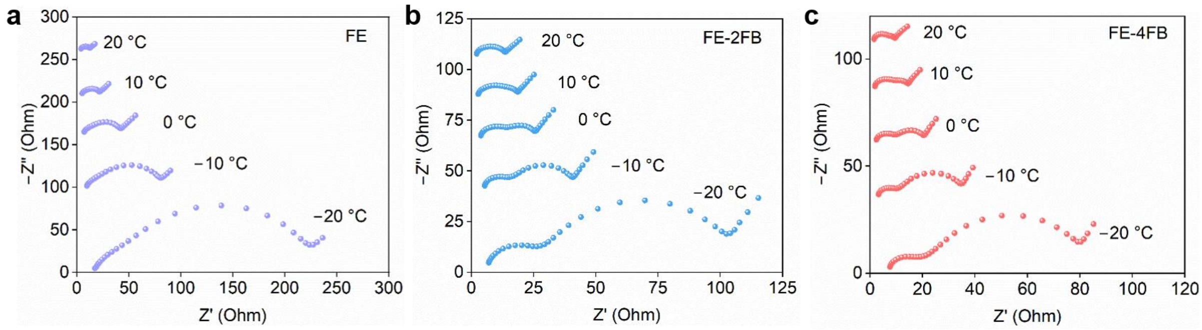

Figure S5. Nyquist plots of Li||Li symmetric cells with (a) FE, (b) FE-2FB and (c) FE-4FB at different temperatures.

Table S2. Ohmic impedance (R_{bulk}), interfacial impedance (R_i), and R_{ct} of the Li||Li cells according to EIS in Figure 1e.

Electrolyte	R_{bulk}	R_i	Rct
FE	18.3	21.2	199.4
FE-2FB	6.9	20.3	82.9
FE-4FB	7.3	13.1	67.5

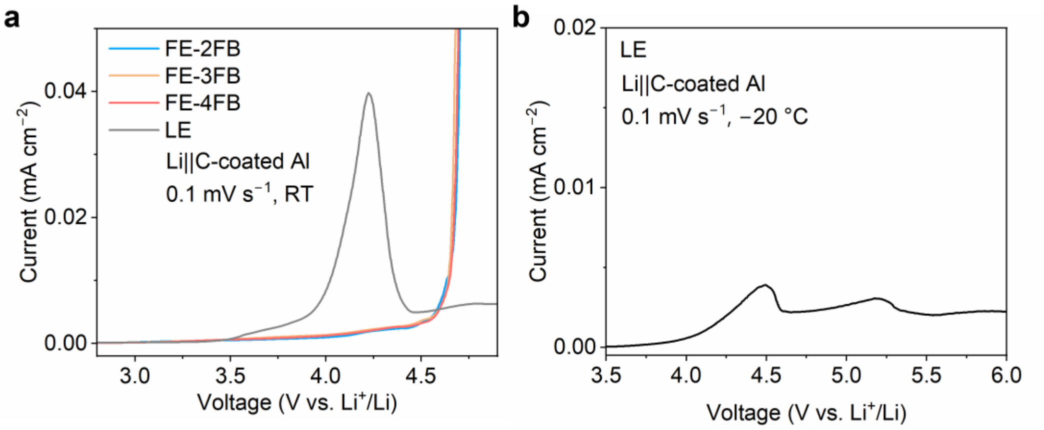

Figure S6. (a) LSV curves of FE-2FB, FE-3FB, FE-4FB, and LE electrolytes at room temperature (RT). (b) LSV curves of LE at low temperature.

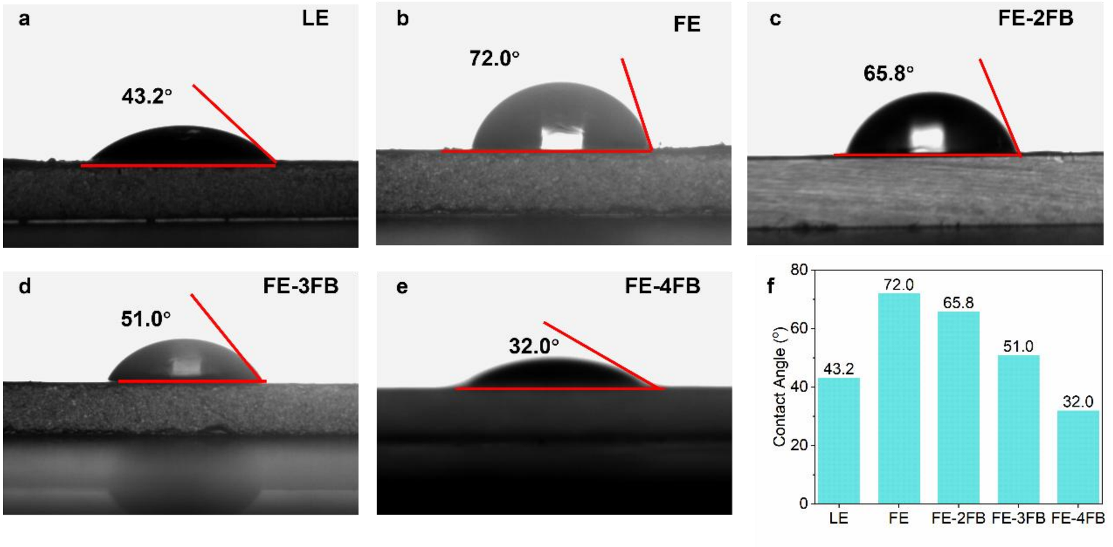

Figure S7. Contact angle measurements of electrolytes on PP separator: (a) LE, (b) FE, (c) FE-2FB, (d) FE-3FB, and (e) FE-4FB. (f) Bar chart comparing the contact angles of different electrolytes.

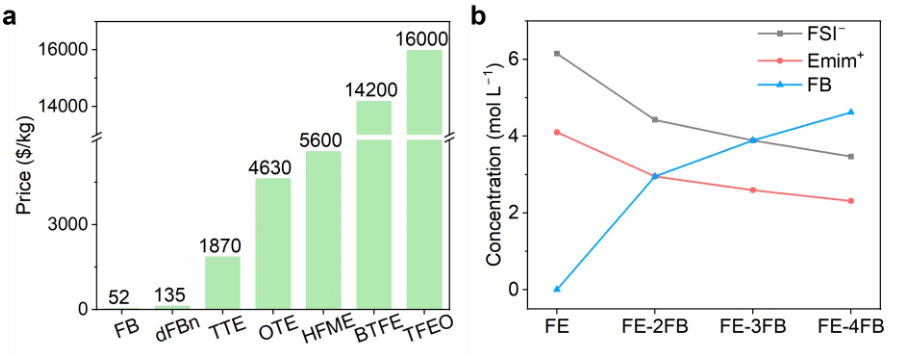

Figure S8. (a) Comparison of market prices of current fluorides. (b) Concentrations of FSI^{-}, $Emim^{+}$, and FB in LCILEs with varying FB content.

Table S3. Computed coordination (CN) numbers of Li^+ with FSI^- and FB in FE-2FB and FE-4FB.

	CN numbers	Probability (Li^+-FB)	Probability (Li^+-FSI^-)
FE-2FB	0	80.24%	0%
	1	17.76%	4.11%
	2	2%	7.67%
	3	0%	34.43%
	4	0%	39.37%
	5	0%	14.42%
	Average number	Li^+-FB: 0.218	Li^+-FSI^-: 3.523
FE-4FB	0	70.29%	0%
	1	24.96%	5.53%
	2	4.06%	11.68%
	3	0.69%	43.18%
	4	0%	37.61%
	5	0%	2%
	Average number	Li^+-FB: 0.351	Li^+-FSI^-: 3.189

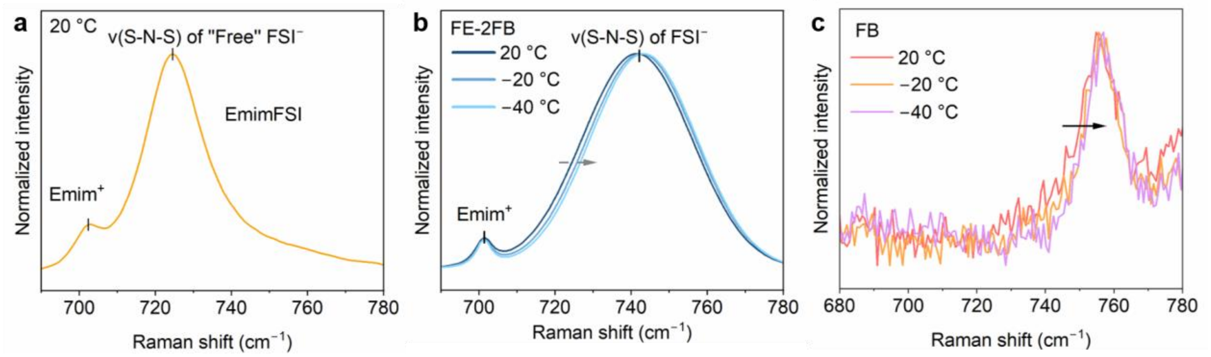

Figure S9. (a) Raman spectrum of EmimFSI at 20 °C. (b) Temperature-dependent Raman spectra of FE-2FB at 20, −20, and −40 °C. (c) Temperature-dependent Raman spectra of FB at 20, −20, and −40 °C.

Note 2: Raman analysis focused on the 690–780 cm^{-1} region, corresponding to the symmetric stretching vibration (v(S–N–S)) of the FSI^- anion. Raman analysis focused on the 750-770 cm^{-1} region, corresponding to FB (**Figure S9a**). At 20 °C, increasing FB content does not affect the peak displacement of LCILEs (**Figure 2e**), indicating that FB interference can be ignored. As temperature decreases, low FB content FE (**Figure 2f**) and FE-2FB (**Figure S9b**) primarily show a rightward shift in the 710-740 cm^{-1} region, which does not overlap with the FB peak, suggesting this shift is unrelated to FB. Additionally, the peak of FB shifts to the right as temperature decreases (**Figure S9c**). In contrast, no peak displacement is observed for high FB content FE-4FB (**Figure 2g**). This further indicates that low-temperature peak displacements in the electrolyte are unrelated to FB. These features support using this region to analyze the Li^+ solvation structure.

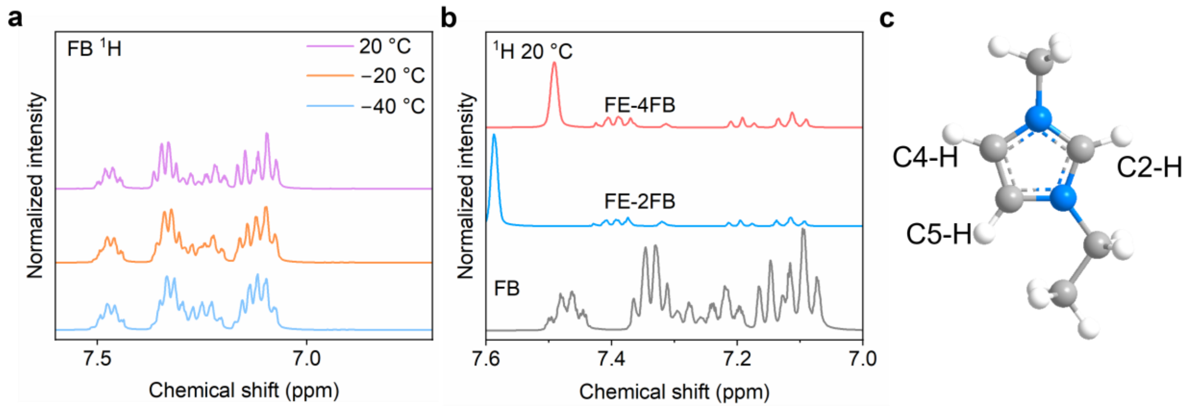

Figure S10. 1D ^1H NMR spectra of (a) FB in the 6.70–7.60 ppm at 20, −20 and −40 °C and (b) FB, FE-2FB, FE-4FB in the 7.00–7.60 ppm region at 20 °C. (c) Chemical structure of $Emim^+$ with the C2–H, C4–H, and C5–H positions marked.

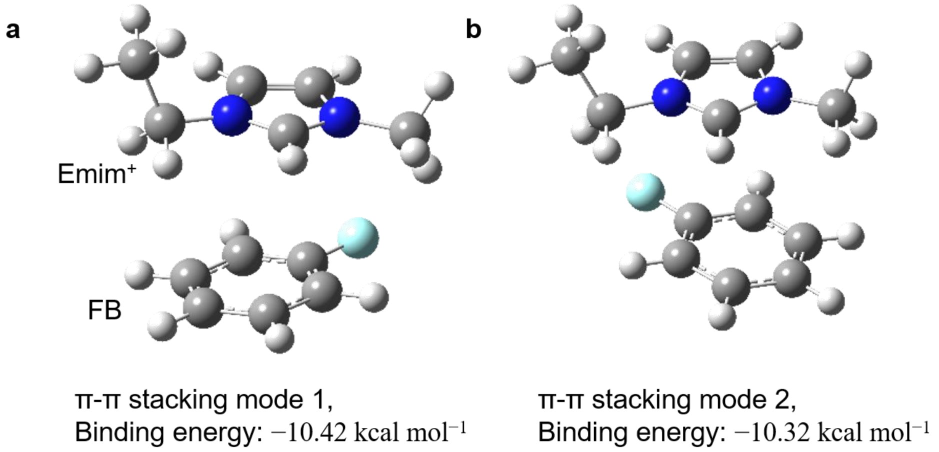

Figure S11. The optimized π-π stacking modes of FB with $Emim^+$ and the corresponding binding energy (N, F, C, and H atoms are represented by blue, green, gray, and white spheres, respectively).

Note 3: The binding energy results from the two π-π stacking modes indicate the presence of this weak interaction. Additionally, the binding energy of π-π stacking mode 1 is similar to that of mode 2, suggesting that both stacking modes coexist in the electrolyte.

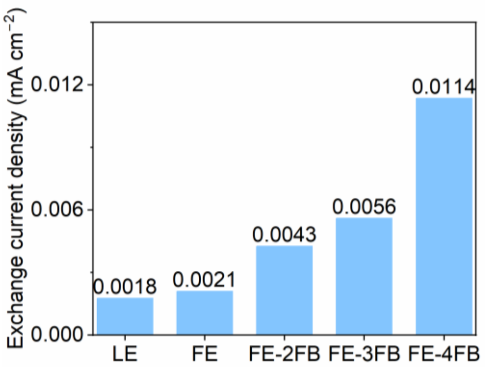

Figure S12. Comparison of exchange current densities of lithium anode in different electrolytes.

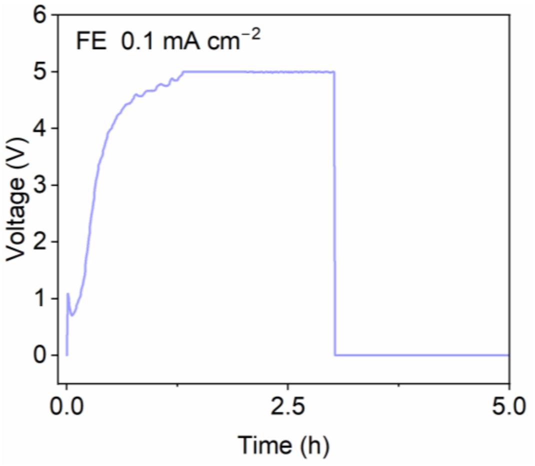

Figure S13. Voltage profiles of a Li||Li symmetric cell with FE electrolyte.

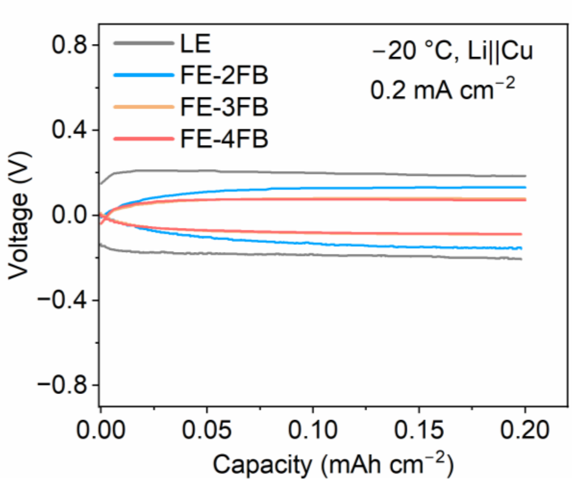

Figure S14. Voltage–capacity curves of Li||Cu cells with FE-2FB, FE-3FB, FE-4FB, and LE electrolytes.

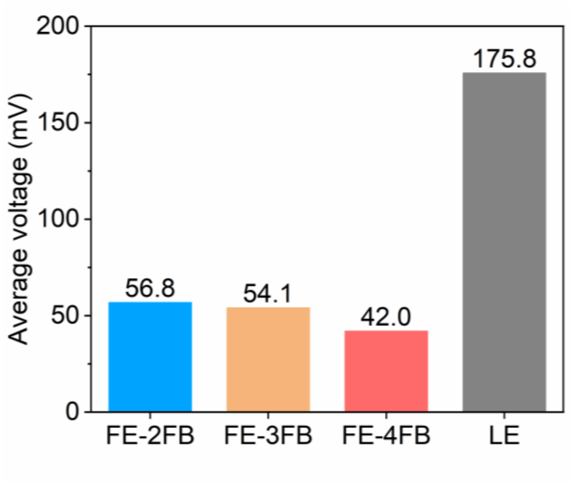

Figure S15. Average voltages Li||Li cells with different electrolytes between 1000th and 2000th cycles shown in Figure 3d.

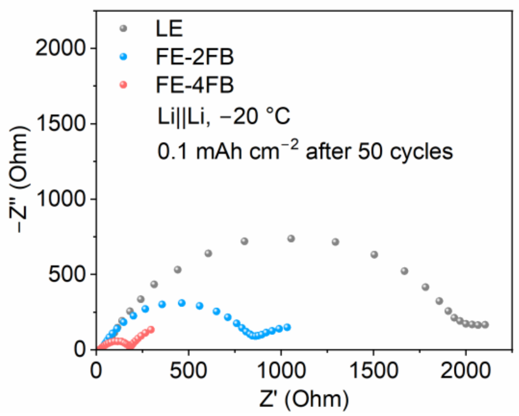

Figure S16. Nyquist plots of Li||Li cells with different electrolytes after 50 cycles at 0.1 mA cm^{-2} with a cycling capacity of 0.1 mAh cm^{-2} at −20 °C.

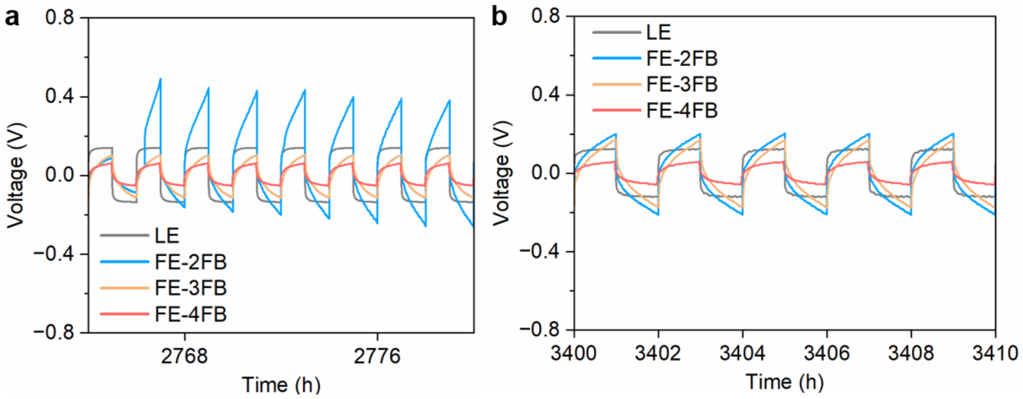

Figure S17. The particle enlarged figure of Figure 3d.

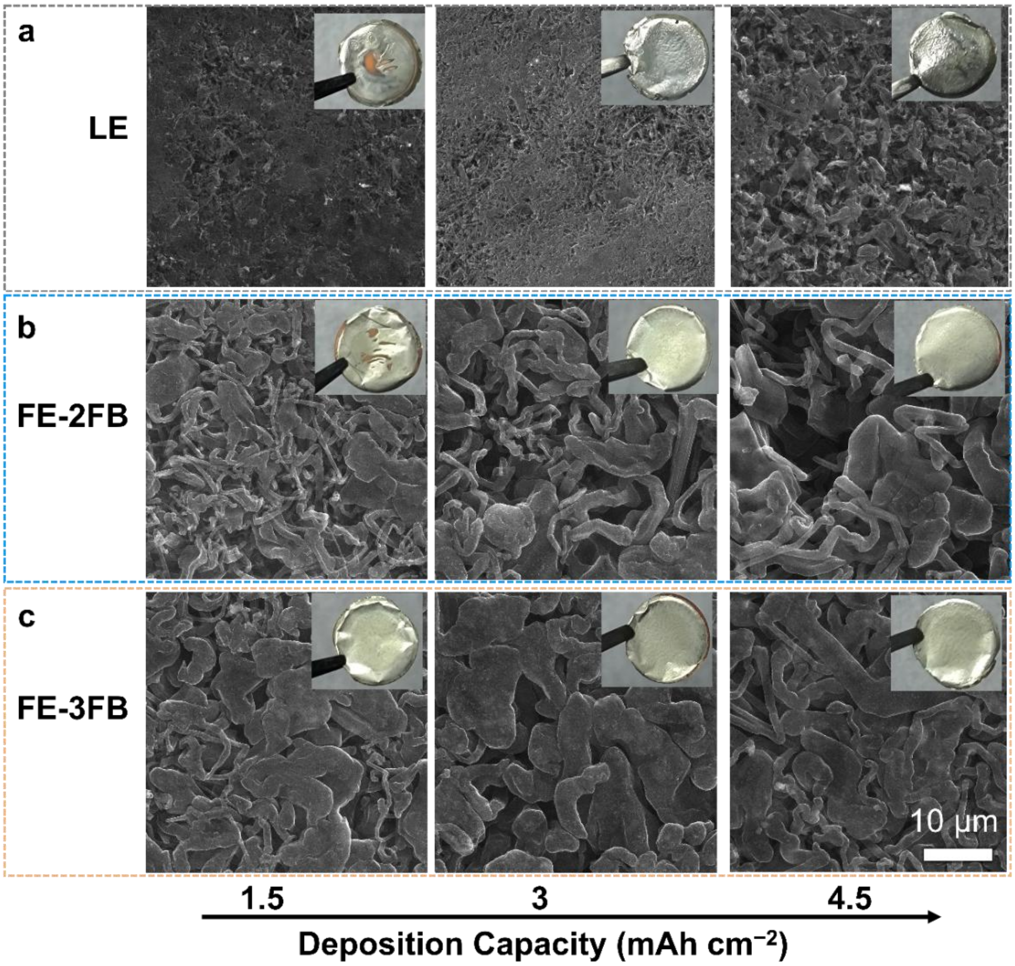

Figure S18. SEM images of lithium deposits on Cu foil at various deposition capacities for Li||Cu cells with different electrolytes at 0.1 mA cm^{-2} (inset: corresponding digital photographs of lithium deposits on Cu foil): (a) LE, (b) FE-2FB, and (c) FE-3FB.

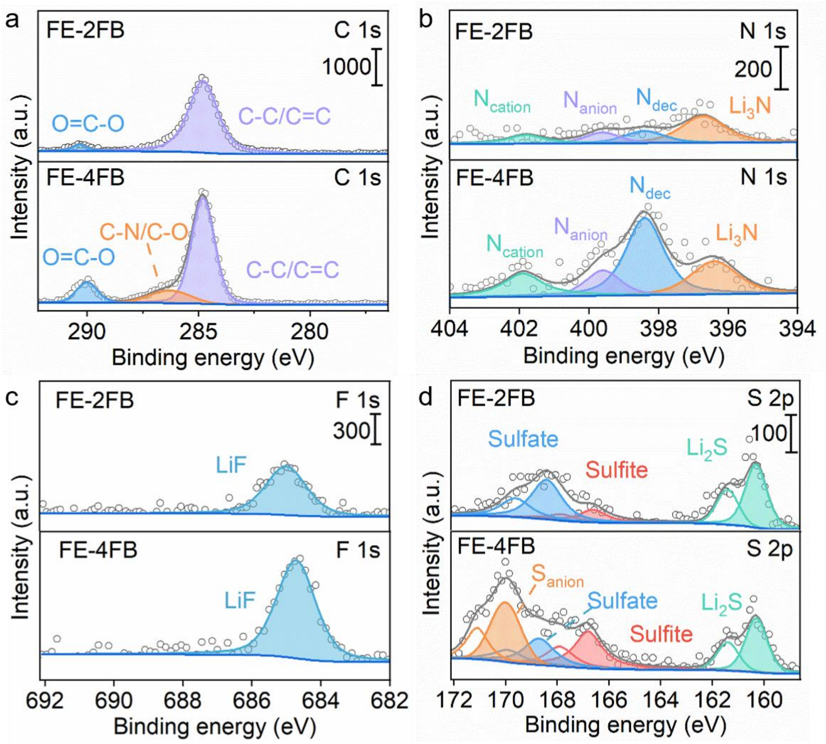

Figure S19. (a) C 1s, (b) N 1s, (c) F 1s and (d) S 2p XPS spectra of cycled lithium metal surface with FE-2FB and FE-4FB electrolytes at 20 °C.

Note 4: Compared to FE-2FB, FE-4FB exhibits a higher ratio of C-C/C=C, LiF, N_{dec}, and Li_3N on the lithium metal surface. This enhancement is primarily attributed to the higher content of FB in FE-4FB and the more complete decomposition of FSI^-, which contributes to stable cycling of lithium metal batteries at room temperature, consistent with results observed at low temperatures. Additionally, the N 1s and S 2p spectra indicate that Li_3N and Li_2S are present in significantly higher proportions within nitrogenous and sulfurous substances than in low-temperature SEI layers (**Figure 4a-f**). This suggests that low temperatures severely hindered the conversion of FSI^-. Additionally, all signals from materials are markedly lower than those observed under low-temperature conditions. This indicates that the inorganic-rich SEI layer formed at room temperature substantially enhances interfacial stability and effectively suppresses continuous decomposition of the electrolyte at the interface.

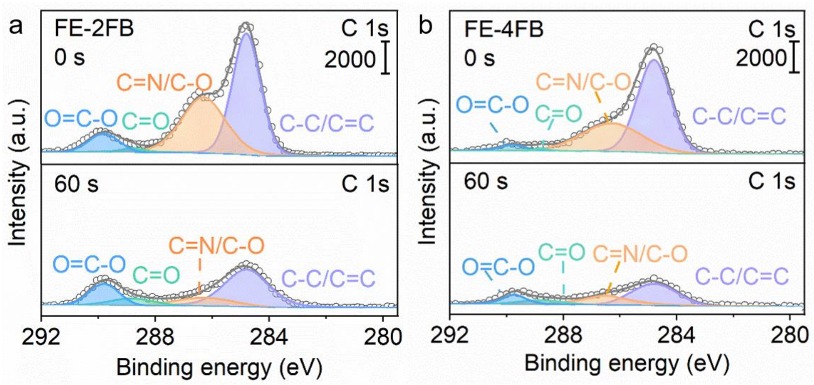

Figure S20. C 1s XPS spectra of the cycled lithium metal surface with (a) FE-2FB and (b) FE-4FB electrolytes at −20 °C.

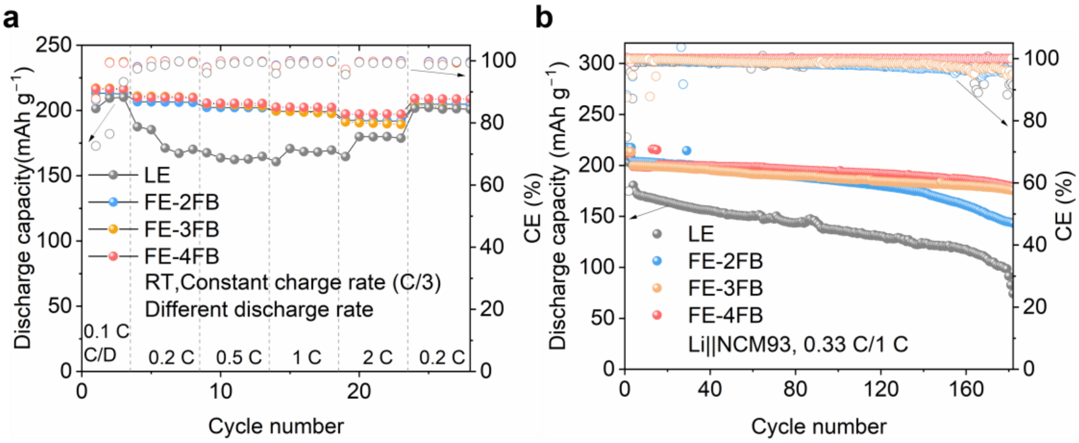

Figure S21. Electrochemical performance of Li||NCM93 cells (NCM93 loading: 10 mg cm^{-2}) with FE-2FB, FE-3FB, FE-4FB, and LE electrolytes at room temperature: (a) rate performances and (b) cycling performance.

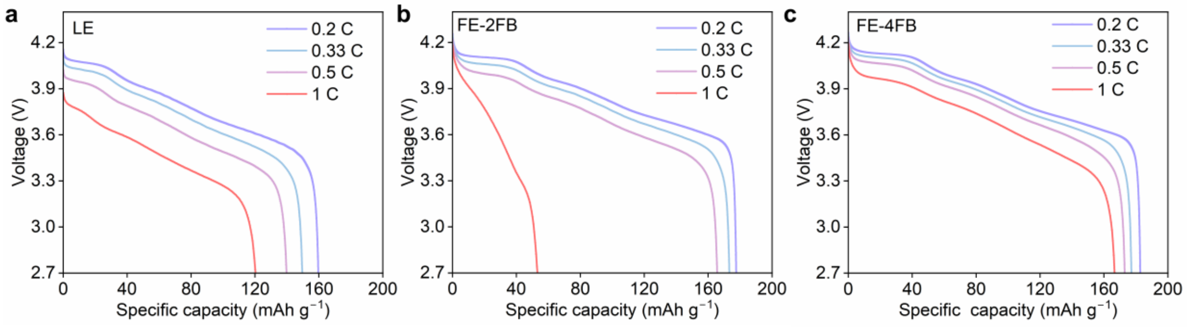

Figure S22. Charge/discharge voltage profiles of cells using (a) LE, (b) FE-2FB, and (c) FE-4FB electrolytes under the cycling conditions described in Figure 5a.

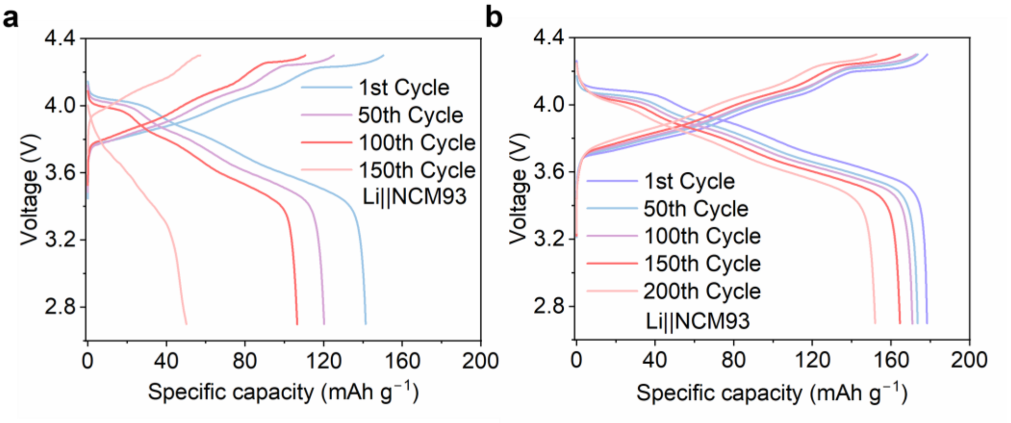

Figure S23. Charge/discharge voltage profiles of cells using (a) LE and (b) FE-4FB under the cycling conditions described in Figure 5c.

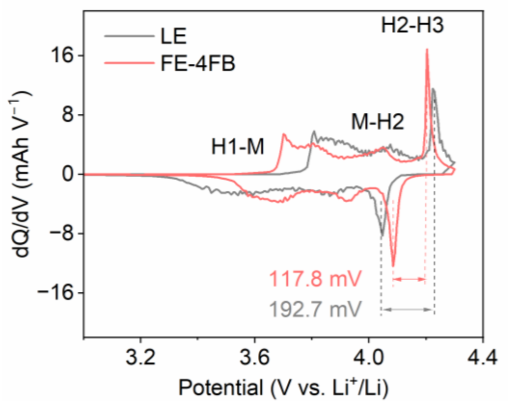

Figure S24. dQ/dV curves of LE and FE-4FB in the first cycle after activation under the testing conditions described in Figure 5c.

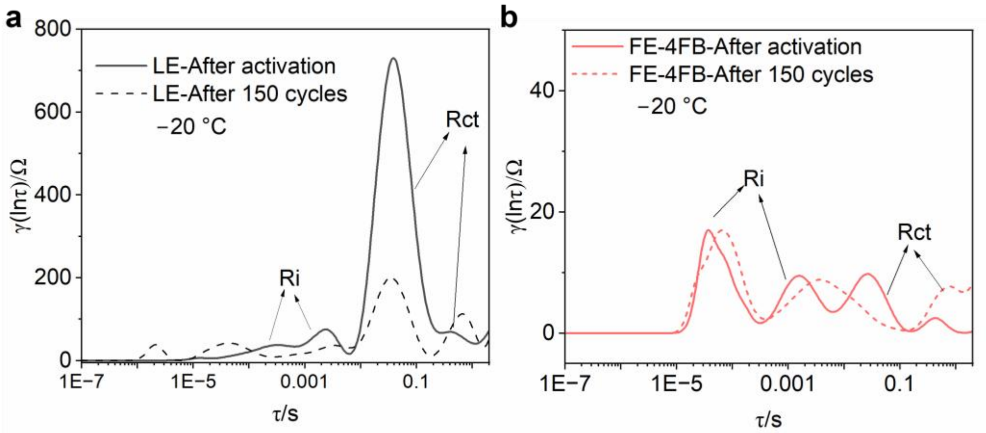

Figure S25. DRT profiles of cell with (a) LE and (b) FE-4FB electrolyte after activation and after 150 cycles under the testing conditions described in Figure 5c.

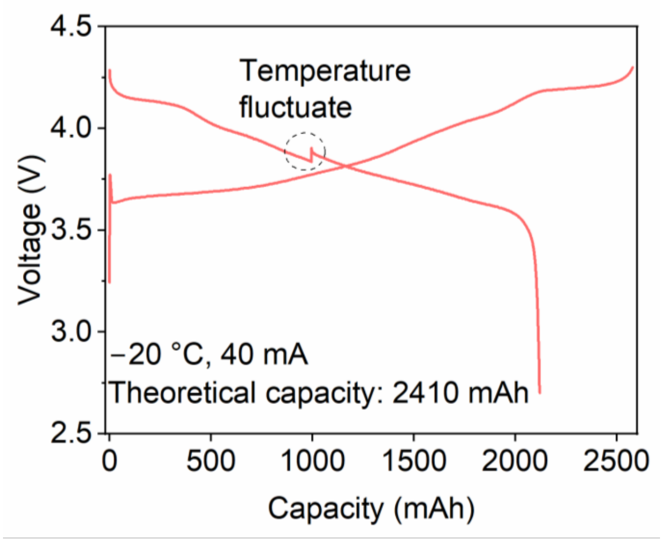

Figure S26. Charge/discharge voltage profile of a pouch cell with FE-4FB at −20 °C.

Table S4. Cell parameters of the Li||NCM93 pouch cell.

NCM93 Cathode	Material	NCM93	NCM93
	Theoretical specific capacity (mAh g^{-1})	200	200
	Mass loading (mg cm^{-2})	10	25
	Area of a single electrode (cm^2)	40.5 (5.20 × 7.78 cm^2)	48.2 (5.20 × 9.26 cm^2)
	Layers	5	5
	Theoretical capacity (mAh)	810	2410
Anode	Material	Li	Li
	Thickness (μm)	60	60
	Layers	6	6
Electrolyte	Quality (g)	5.4	9.2
	E/C ratio (g Ah^{-1})	6.67	3.82

Reference

(S1) Wang, Z.; Gu, X.; Zhu, J.; et al. Weakly-Solvated and Co-Intercalation-Free Ether-Based Electrolytes Enhance the Low- Temperature and Fast-Charging Performance of LiFePO4||Graphite Batteries. *Angewandte Chemie International Edition* 2026, *65* (1), e21171. https://doi.org/10.1002/anie.202521171.

(S2) Zhao, Z.; Wang, A.; Chen, A.; et al. Leveraging Ion Pairing and Transport in Localized High-Concentration Electrolytes for Reversible Lithium Metal Anodes at Low Temperatures. *Angewandte Chemie International Edition* 2024, *63* (45), e202412239. https://doi.org/10.1002/anie.202412239.

(S3) Abraham, M. J.; Murtola, T.; Schulz, R.; et al. GROMACS: High Performance Molecular Simulations through Multi-Level Parallelism from Laptops to Supercomputers. *SoftwareX* 2015, 1–2, 19–25. https://doi.org/10.1016/j.softx.2015.06.001.

(S4) Van Der Spoel, D.; Lindahl, E.; Hess, B.; et al. GROMACS: Fast, Flexible, and Free. *Journal of Computational Chemistry* 2005, *26* (16), 1701–1718. https://doi.org/10.1002/jcc.20291.

(S5) Berendsen, H. J. C.; van der Spoel, D.; van Drunen, R. GROMACS: A Message-Passing Parallel Molecular Dynamics Implementation. *Computer Physics Communications* 1995, *91* (1), 43–56. https://doi.org/10.1016/0010-4655(95)00042-E.

(S6) Jorgensen, W. L.; Maxwell, D. S.; Tirado-Rives, J. Development and Testing of the OPLS All-Atom Force Field on Conformational Energetics and Properties of Organic Liquids. *Journal of the American Chemical Society* 1996, *118* (45), 11225–11236. https://doi.org/10.1021/ja9621760.

(S7) Kaminski, G. A.; Friesner, R. A.; Tirado-Rives, J.; et al. Evaluation and Reparametrization of the OPLS-AA Force Field for Proteins via Comparison with Accurate Quantum Chemical Calculations on Peptides. *The Journal of Physical Chemistry B* 2001, *105* (28), 6474–6487. https://doi.org/10.1021/jp003919d.

(S8) Hess, B.; Bekker, H.; Berendsen, H. J. C.; et al. LINCS: A Linear Constraint Solver for Molecular Simulations. *Journal of Computational Chemistry* 1997, *18* (12), 1463–1472. https://doi.org/10.1002/(SICI)1096-987X(199709)18:12<1463::AID-JCC4>3.0.CO;2-H.

(S9) Tian L. Molclus program, Version 1.9.9.9, http://www.keinsci.com/research/molclus.html (accessed 11 24, 2022)

(S10) Bannwarth, C.; Ehlert, S.; Grimme, S. GFN2-xTB—An Accurate and Broadly Parametrized Self-Consistent Tight-Binding Quantum Chemical Method with Multipole Electrostatics and Density-Dependent Dispersion Contributions. *Journal of Chemical Theory and Computation* 2019, *15* (3), 1652–1671. https://doi.org/10.1021/acs.jctc.8b01176.

(S11) Bannwarth, C.; Caldeweyher, E.; Ehlert, S.; et al. Extended Tight-Binding Quantum Chemistry Methods. *Wiley Interdisciplinary Reviews: Computational Molecular Science* 2021, *11* (2), e1493. https://doi.org/10.1002/wcms.1493.

(S12) Frisch, M. J.; Trucks, G. W.; Schlegel, H. B.; et al. Gaussian 16, Revision C.01, Gaussian, Inc., Wallingford CT, 2019. https://gaussian.com

(S13) Grimme, S.; Ehrlich, S.; Goerigk, L. Effect of the Damping Function in Dispersion Corrected Density Functional Theory. *Journal of Computational Chemistry* 2011, *32* (7), 1456–1465. https://doi.org/10.1002/jcc.21759.

(S14)Weigend, F.; Ahlrichs, R. Balanced Basis Sets of Split Valence, Triple Zeta Valence and Quadruple Zeta Valence Quality for H to Rn: Design and Assessment of Accuracy. *Physical Chemistry Chemical Physics* 2005, *7* (18), 3297–3305. https://doi.org/10.1039/b508541a.
(S15)Schäfer, A.; Horn, H.; Ahlrichs, R. Fully Optimized Contracted Gaussian Basis Sets for Atoms Li to Kr. *The Journal of Chemical Physics* 1992, *97* (4), 2571–2577. https://doi.org/10.1063/1.463096.
(S16)Schäfer, A.; Huber, C.; Ahlrichs, R. Fully Optimized Contracted Gaussian Basis Sets of Triple Zeta Valence Quality for Atoms Li to Kr. *The Journal of Chemical Physics* 1994, *100* (8), 5829–5835. https://doi.org/10.1063/1.467146.
